# Dual ifgMosaic: A Versatile Method for Multispectral and Combinatorial Mosaic Gene-Function Analysis

**DOI:** 10.1016/j.cell.2017.07.031

**Published:** 2017-08-10

**Authors:** Samuel Pontes-Quero, Luis Heredia, Verónica Casquero-García, Macarena Fernández-Chacón, Wen Luo, Ana Hermoso, Mayank Bansal, Irene Garcia-Gonzalez, Maria S. Sanchez-Muñoz, Juan R. Perea, Adrian Galiana-Simal, Iker Rodriguez-Arabaolaza, Sergio Del Olmo-Cabrera, Susana F. Rocha, Luis M. Criado-Rodriguez, Giovanna Giovinazzo, Rui Benedito

**Affiliations:** 1 Molecular Genetics of Angiogenesis Group, Centro Nacional de Investigaciones Cardiovasculares Carlos III (CNIC), Melchor Fernández Almagro, 3, Madrid, E-28029, Spain; 2 Transgenesis Unit, Centro Nacional de Investigaciones Cardiovasculares Carlos III (CNIC), Melchor Fernández Almagro, 3, Madrid, E-28029, Spain; 3 Pluripotent Cell Technology Unit, Centro Nacional de Investigaciones Cardiovasculares Carlos III (CNIC), Melchor Fernández Almagro, 3, Madrid, E-28029, Spain

**Keywords:** mosaic, epistasis, transgenesis, Brainbow, Confetti, Rosa26, Notch, VEGF, angiogenesis

## Abstract

Improved methods for manipulating and analyzing gene function have provided a better understanding of how genes work during organ development and disease. Inducible functional genetic mosaics can be extraordinarily useful in the study of biological systems; however, this experimental approach is still rarely used in vertebrates. This is mainly due to technical difficulties in the assembly of large DNA constructs carrying multiple genes and regulatory elements and their targeting to the genome. In addition, mosaic phenotypic analysis, unlike classical single gene-function analysis, requires clear labeling and detection of multiple cell clones in the same tissue. Here, we describe several methods for the rapid generation of transgenic or gene-targeted mice and embryonic stem (ES) cell lines containing all the necessary elements for inducible, fluorescent, and functional genetic mosaic (ifgMosaic) analysis. This technology enables the interrogation of multiple and combinatorial gene function with high temporal and cellular resolution.

## Highlights

• Dual ifgMosaic enables the study of the function of multiple genes in the same tissue
• Imaging of up to 15 different cell populations expressing a unique set of genes
• Dual ifgMosaic enables combinatorial epistasis analysis at single-cell resolution
• Open-source DNA engineering strategy that simplifies the generation of genetic mosaics

## Graphical Abstract

**Figure:**
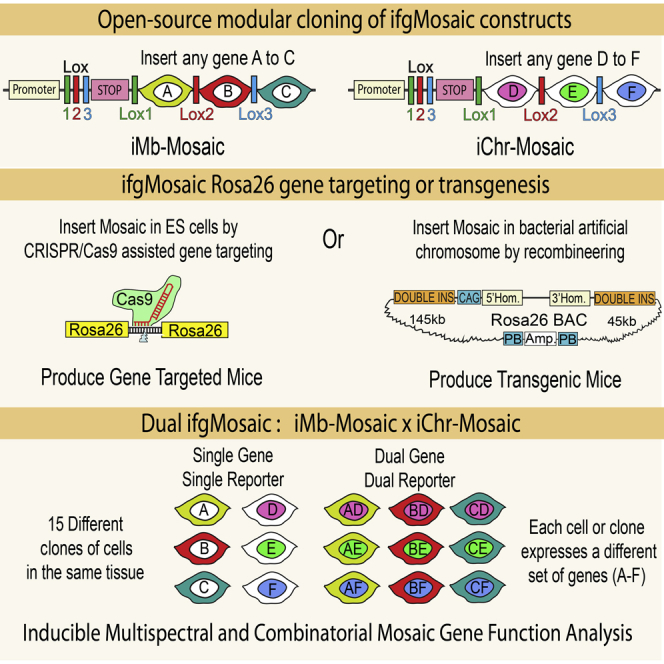

## Teaser

Multiple strategies are developed to enable multispectral and combinatorial mosaic gene-function analysis in mice, allowing comparison of molecular phenotypes on a cell-by-cell basis.

## Introduction

The ability to modify gene function at very high temporal and spatial resolution has radically altered the study of biologically and biomedically relevant processes. Genetic mosaics are a particularly powerful research tool because they allow the study of cell-autonomous effects when distinct mutant and wild-type cells confront the same environment in the same tissue or organism. The analysis of genetic mosaics allows single-cell, or clonal phenotypic analysis, where the only difference between the cells used for comparative analysis is the induced mutation or the expression of a given gene in an otherwise identical organism and genetic background. This approach is often more precise and informative than the use of classical genetics, in which the comparison is made between distinct wild-type and mutant animals that may develop secondary and non-cell-autonomous phenotypes over time, distorting interpretation of the process under study. Genetic mosaics have been used extensively in the fruit fly due to the ease of performing mitotic recombination and have revolutionized the study of cell biology in this model organism. However, it is technically more challenging to induce and analyze genetic mosaics in the mouse, the most widely used model organism in biomedical research. One of the methods used to induce mitotic genetic mosaics in mice is MADM (mosaic analysis with double markers), which allows the labeling of control and mutant cells with different markers (Zong et al., 2005). However, this method relies on very rare interchromosomal recombination events, leading to the generation of only a few clones of labeled control and mutant cells in the tissue. The generation of these genetic mosaics also cannot be accurately controlled in time, since these rare events can only occur with constitutively active Cre lines and not tamoxifen-inducible CreERT2 lines, which are weaker and only transiently active. In addition, the requirement for genetic linkage between the engineered MADM elements and another gene mutation means that currently this method can only be performed with genes located on 4 of the 20 mouse chromosomes (Zong, 2014). A more widely used method to generate functional genetic mosaics in the mouse involves partial or mosaic induction of Cre-*LoxP* intrachromosomal recombination, resulting in deletion of floxed genes. With this method, the location, timing, and frequency of the recombination events can be regulated by restricting the expression of CreERT2 to a given tissue and by varying the timing and dosage of the CreERT2 ligand tamoxifen. Mosaic induction of floxed-alleles recombination is frequently associated with the use of independent, and genetically distinct, fluorescent reporters of recombination, or Cre activity (Srinivas et al., 2001). However, several studies have shown that these cannot be used to reliably report another given gene deletion or activation (Liu et al., 2013, Long and Rossi, 2009, Vooijs et al., 2001). This lack of correlation between recombination of a reporter allele, and alteration of the gene of interest, means that the majority of current conditional and mosaic genetic modifications and function analysis in the mouse are conducted without a reliable readout. This technical problem can be circumvented by immunostaining for the protein encoded by the deleted or activated gene, to ensure that it is either absent or upregulated in the desired cells. However, for most proteins, the immunostaining signal is too weak or does not provide sufficient cellular resolution to clearly identify the cell shape and thus permit quantification of the phenotype of cells with a given genetic alteration. Moreover, immunostaining requires fixed cells and is thus incompatible with direct live imaging of the mutant or recombined cells.

With this in mind, we have developed and tested new strategies for the conditional induction of mosaic gene expression linked to the expression of different and compatible fluorescent marker proteins. The methods described here use an open-source DNA engineering strategy that greatly simplifies the production of large and complex constructs for inducible, fluorescent, and genetic mosaic (ifgMosaic) studies. We also provide an easy-to-follow pipeline for mouse *Rosa26* BAC recombineering and transgenesis that enables robust and rapid generation of *ifgMosaic* mice and a method for CRISPR/Cas9-induced gene targeting of large mosaic constructs in the *Rosa26* locus of mouse embryonic stem (ES) cells. This methodology will greatly simplify combinatorial mosaic gene-function analysis with high genetic and cellular resolution.

## Results

### Dual ifgMosaic Strategy for High-Resolution Mosaic Analysis of Gene Function

**Figure 1:**
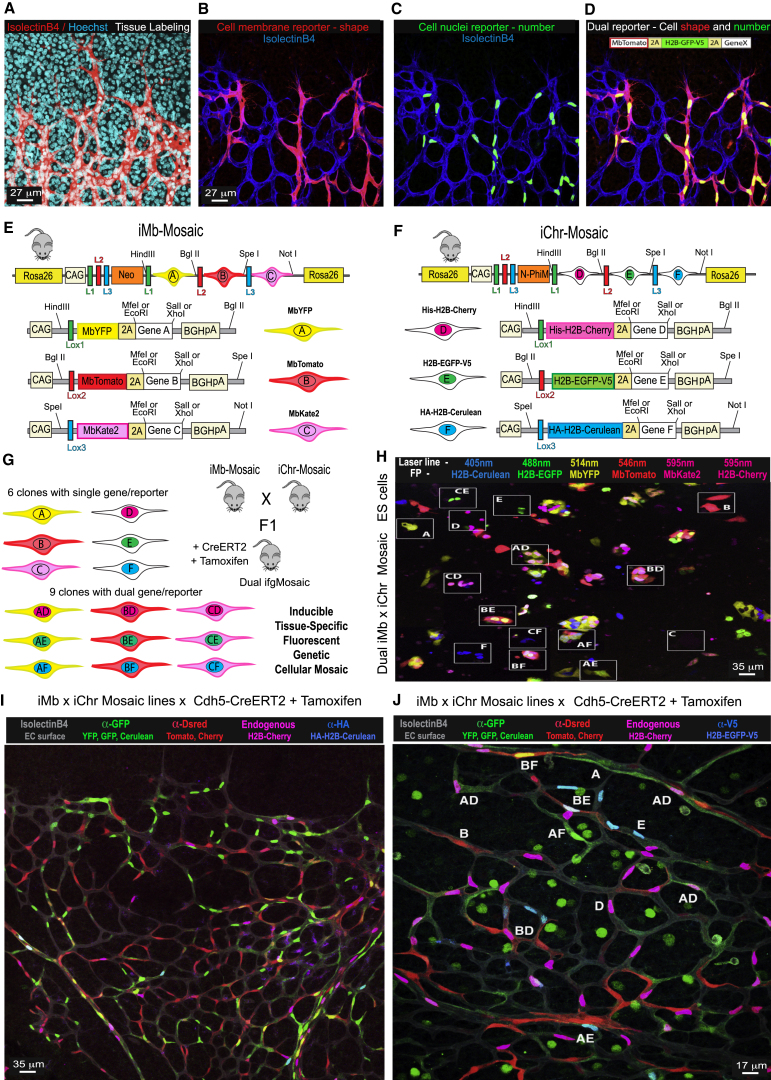
Inducible Dual Membrane and Chromatin Mosaic Constructs, Cells, and Mice (A) Endothelial surface (IsolectinB4) and DNA (Hoechst) markers allow the visualization of tissue architecture but not single cells. (B–D) The cell membrane (B) or nuclei (C) can be visualized with Mb or H2B-tagged reporter FPs, but only dual labeling (D) allows the full identification of a cell shape and number. (E and F) In *iMb-Mosaic* and *iChr-Mosaic* constructs and mouse lines, recombination is only possible between identical Lox (L) sites (L1, L2, or L3). Therefore, Cre-mediated recombination generates three possible outcomes for each construct (A, B, C or D, E, F) that are mutually exclusive in any single cell. See Figure S2 for the DNA elements description. (G) When one mouse or cell line contains the two inducible mosaic alleles (*iMb-Mosaic* and *iChr-Mosaic*), up to 15 different cell clones can be induced in the same tissue, allowing combinatorial epistasis analysis at single-cell resolution. A–F indicate the single or dual color code for the clone. (H) *Dual ifgMosaic* in ES cells and live imaging of their distinct fluorescent signals with different laser lines. See also Figure S2J. (I and J) Retina vasculature of a growing newborn mouse containing the *iMb-Mosaic*, *iChr-Mosaic*, and *Cdh5(PAC)-CreERT2* alleles, 3 days after induction of recombination with tamoxifen. The genetic mosaic is detected within the IsolectinB4+ vascular tissue by scanning the endogenous fluorescence and immunostaining signals. Single- and dual-labeled clones are visible at low (I) and higher (J) magnification.

**Figure S1:**
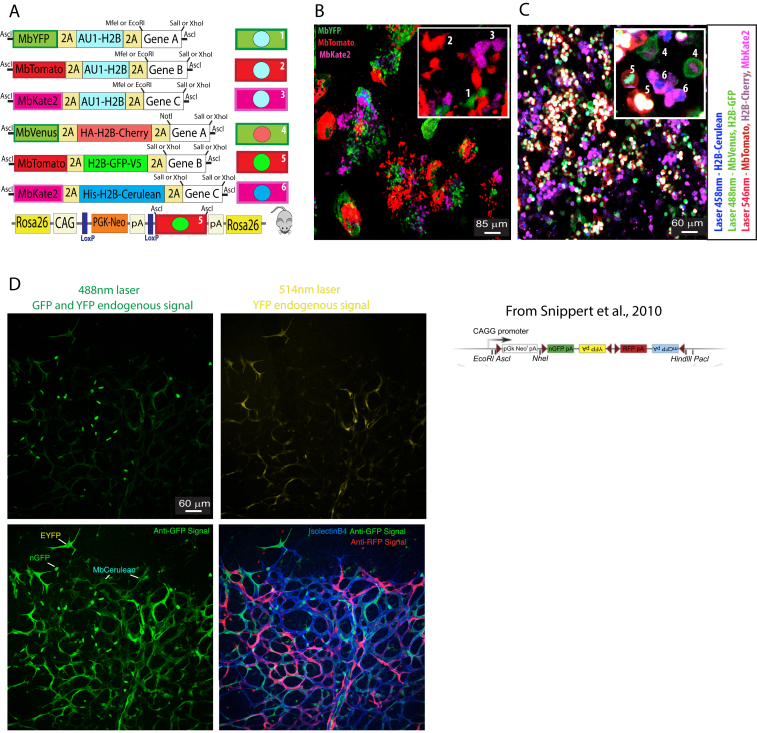
Multiple Dual Fluorescent Reporters for Determining Gene Effects on Cell Shape, Migration, and Proliferation, Related to Figure 1 (A) Several different constructs (1-6) were assembled and tested for the simultaneous expression of Mb- and H2B-tagged marker proteins and a given gene of interest. (B) Immunostaining of stable cell lines expressing constructs 1-3 with three different antibodies (anti-GFP, anti-DsRed, and anti-Kate2). (C) Live imaging (see also Movie S1) with 3 laser lines (458, 488, and 546nm) and 5 different detector settings to capture the chromatin and membrane of all cells containing constructs 4-6 (see, a), allowing simultaneous visualization of the effect of different genes on cell division and migration in the same acquisition field. Legend: Mb - Tag that localizes proteins to the cell membrane. 2A – 21-aminoacid viral peptide sequence cleaved at the C-terminal position, allowing equimolar and independent localization of two flanking proteins coded in the same ORF. Au1, V5, HA, His - short epitope tags that can be detected by specific antibodies. H2B - Histone 2B protein tag that localizes proteins to the chromatin/cell nucleus. pA - polyadenylation transcription stop signal. (D) Brainbow 2.1 expression in endothelial cells of the mouse retina. This mouse line allows the induction after Cre recombination of up to 4 FPs (nGFP, YFP, MbCFP, and RFP) that are excited and emit signals at different wavelengths. Confocal scanning of the endogenous protein fluorescence signals after tissue fixation can only provide high resolution of the strongest signals. Immunostaining with antibodies to GFP greatly improves signal intensity, but detects nGFP, YFP, and MbCFP simultaneously, compromising their proper distinction in the same tissue.

One of the difficulties limiting our understanding of biological processes is our inability to clearly distinguish phenotypes at the single-cell level. Most tissues are composed of groups of tightly packed and adhered cells. Classical mouse genetics and standard antibody immunostaining provide tissue resolution but not single-cell resolution (Figure 1A). Standard unicolor or single-molecule reporters, which label a given cell or tissue with a single protein localized in the cytoplasm, membrane, or nucleus, do not allow the simultaneous and accurate determination of clone-cell shape and number, thus limiting our understanding of the clonal phenotype and its tissue distribution (Figures 1B and 1C). We therefore assembled several distinct DNA constructs that allow conditional and simultaneous expression of two distinct membrane- or chromatin-localized reporters and a gene of interest in the same recombined cells (Figures 1D and S1A). This approach increases the cellular resolution and the quantitative power of clonal functional analysis because cell shape and number can both be quantified by immunostaining or live imaging, allowing highly accurate tracking of the mutant-cell morphology, migration, and proliferation (Figures S1B and S1C; Movie S1). However, an inherent limitation of this strategy for labeling cells with a given gene expression is that although it allows us to visualize and quantify the shape and number of cells expressing our gene of interest, we cannot see the adjacent non-recombined wild-type cells at the same resolution (Figure 1D). Therefore, this strategy does not allow proper control of the phenotype caused by the genetic induction, since it is not possible to appreciate local phenotypic differences between mutant and control or wild-type cells. To overcome these limitations, and be able to induce and label cell clones with distinct gene expression in the same tissue *in vivo*, we adapted a strategy based on multiple and mutually exclusive *LoxP* sites that was previously used to generate the Brainbow and Confetti mouse lines (Livet et al., 2007, Snippert et al., 2010). With this approach, it is possible to induce multicolor labeling and fate map different cells in a tissue expressing Cre or CreERT2. However, existing DNA constructs and mouse lines do not allow simultaneous tracking of a cell’s nucleus and membrane; moreover, due to the closed DNA engineering strategy used, existing constructs also do not allow the insertion and mosaic co-expression of other genes of interest. In some of the existing mouse lines, the expression of the different fluorescent proteins (FPs) cannot be distinguished by immunostaining (Figure S1D) because they are derived from the same species (like YFP, CFP, GFP) and thus have no unique epitopes.

**Figure S2:**
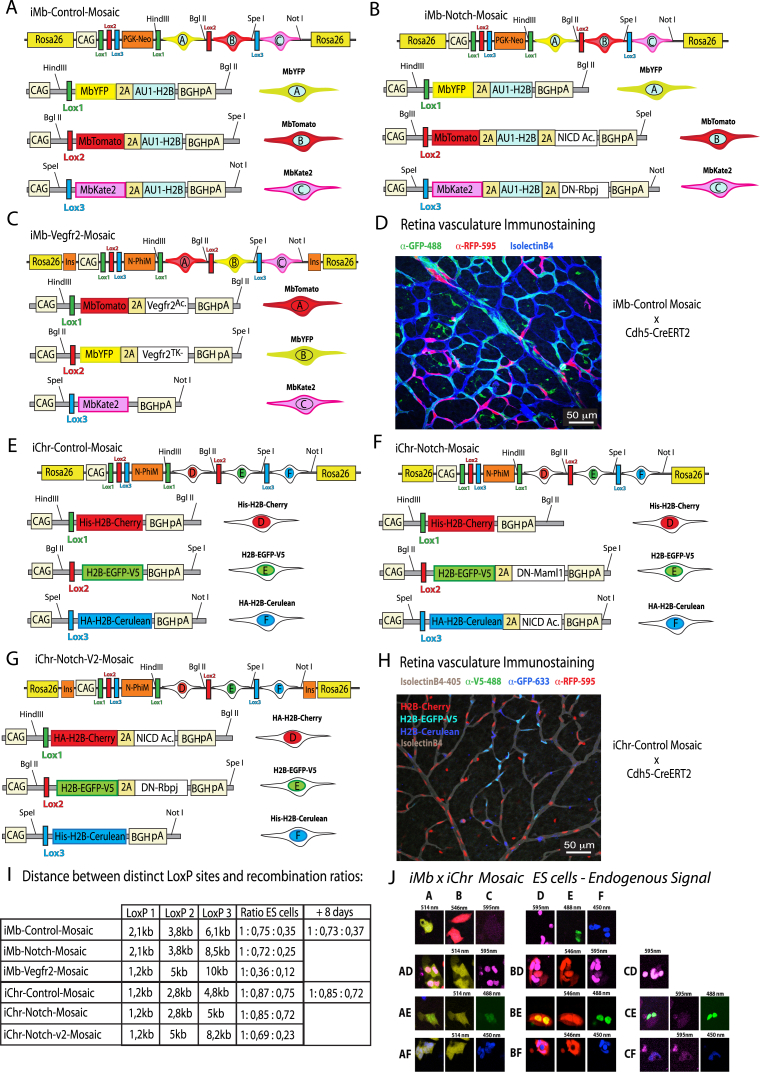
Inducible Membrane and Chromatin Mosaic Constructs, Cells, and Mice, Related to Figures 1–4 (A–C) Some of the *iMb-Mosaic* DNA constructs assembled and used to generate ES cell and mouse lines and the indicated abbreviated names. CAG, Strong and ubiquitous promoter; PGK-Neo, resistance marker for ES cell selection; L1, *LoxN*; L2, *Lox2272*; L3, *LoxP*; 2A, viral peptide allowing equimolar expression of multiple independent proteins from a single ORF; Mb, membrane tag; HA, V5 and His (small epitopes that can be used for specific antibody detection); H2B, histone tag that targets proteins to the chromatin/nucleus; BghpA, bovine growth hormone polyadenylation signal to stop transcription; N-PhiM, non-fluorescent protein that is used as a reporter of promoter expression. DN-Rbpj and DN-Maml1 are dominant-negative proteins that sequester the endogenous Notch intracellular domain (NICD) and reduce Notch receptor signaling in a cell-autonomous manner. NICD Ac. or NICD-PEST is the active form of NICD, containing the native PEST domain that results in a relatively moderate increase of ligand independent Notch activity. VEGFR2^Ac.^ is the constitutively active form of VEGFR2 without the extracellular domain. *Vegfr2*^*TK−*^ is a tyrosine kinase-domain mutant version of murine *Vegfr2* that strongly reduces VEGF signaling in a cell-autonomous manner. (D) Representative confocal micrograph of a retina from a mouse containing the alleles *iMb-Control-Mosaic* and *Cdh5-CreERT2*, which results in a mosaic of ECs (IsolectinB4+) expressing MbYFP or MbTomato or MbKate2. (E–G) Some of the *iChr-Mosaic* DNA constructs assembled and used to generate ES cell and mouse lines and the indicated abbreviated names. (H) Representative confocal micrograph of a retina from a mouse containing the alleles *iChr-Control-Mosaic* and *Cdh5-CreERT2*, which results in a mosaic of ECs (IsolectinB4+), expressing H2B-Cherry, H2B-GFP-V5, or H2B-Cerulean. Cerulean is detected by the anti-GFP antibody only. (I) For each construct is indicated the distinct inter-*LoxP* sites genetic distances and the detected cellular ratios in ES cells 48h (left) or 8 days (right) after transfection with Cre-expressing plasmids. Ratios in *iMb-Mosaic* indicate relative surface area, whereas in *iChr-Mosaic* indicate relative cell number. At least two independent Cre plasmid transfection experiments and 12 pictures representing large microscopic fields (10x lens) were used to calculate the indicated mean values. (J) Confocal scanning micrographs of selected areas shown in Figure 1H, showing the different fluorescence signals from an ES cell line containing both the *iMb-Control-Mosaic* and *iChr-Control-Mosaic* alleles. The color combinations make it possible to separate multiple cell clones. The MbKate2 signal from the *iMb-Control-Mosaic* allele is weak when acquired with the same confocal acquisition settings used for the stronger H2B-Cherry signal from the *iChr-Control-Mosaic* allele.

Using inducible functional genetic mosaics to gain insight into biological processes occurring in the mouse requires a high level of retrospective clonal and phenotypic resolution in order to infer with high confidence how the mosaic cellular phenotype developed over time. For this, the endpoint clonal complexity/resolution needs to be high and clearly detectable by direct imaging or immunostaining in order to obtain statistically significant data. However, unlike simple fluorescent mosaics, to increase the clonal resolution of functional genetic mosaics, we cannot rely on combinatorial recombination of multicopy transgenes, like in Brainbow mice (Livet et al., 2007), since it would generate a mix of cells with combinatorial and unknown multiple gene expression levels in a single cell. To significantly increase cell, clone, and gene-function resolution, we designed a strategy based on the induced recombination of just two types of *ifgMosaic* constructs inserted in the *Rosa26* locus (Figures 1E, 1F, and S2). With these constructs, we generated two types of mouse lines. One allows the inducible expression of three membrane-localized FPs (*iMb-Mosaic*) and the other the induction of three chromatin-localized FPs (*iChr-Mosaic*), each with or without the co-expression of other genes of interest. In ES cells or F1-generation mice carrying both the *iMb-Mosaic* and the *iChr-Mosaic* alleles, we can induce different combinations of recombination to generate up to nine different cell clones with dual labeling of the membrane (for cell shape) and chromatin/nuclei (for cell unit count and proliferation analysis) and six other clones with single membrane or nuclear labeling (Figures 1G and 1H). Thus, a total of 15 clones or up to six genes can be induced in a stochastic/individual or combinatorial manner, allowing study of their individual or combined function in different cells of the same tissue. Contrasting the single probabilistic and non-parallel recombination obtained with classical genetics (Liu et al., 2013), the Dual iMb-Mosaic x iChr-Mosaic (Dual ifgMosaic) strategy (Figures 1I and 1J) can support higher recombination rates without compromising cellular, clonal, or functional resolution because the probability of obtaining a given dual color code is low and will correspond with 100% fidelity, due to the viral 2A peptide (Trichas et al., 2008), to the defined equimolar co-expression of one or two genes of interest. The Dual ifgMosaic strategy is especially suited to genetic epistasis analysis at single-cell or clonal resolution. The detection of adjacent cell clones expressing different combinations of genes and marker proteins in the same tissue and field of view allows direct measurements and phenotypic comparisons in the same environmental context, a crucial step toward understanding the precise role of genes in the cells forming a tissue. By developing an open-source and high-throughput DNA engineering strategy, we were able to rapidly generate several *iMb-* and *iChr-Mosaic* constructs and mouse lines (Figure S2). With some of these lines, it is only possible to induce the expression of FPs, which serve as experimental mosaic controls, or provide a way of increasing clonal complexity and resolution, whereas in other lines, the expression of the different FPs is linked to the expression of specific genes that modulate the function of different signaling pathways.

### Characterization of *iChr-Notch-Mosaic* and *iMb-Vegfr2-Mosaic* ES Cells and Mice

**Figure 2:**
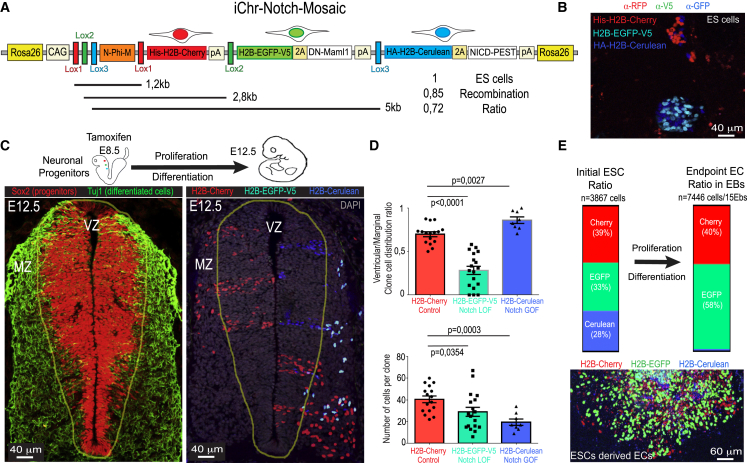
Inducible *iChr-Notch-Mosaic* ES Cells and Mice (A) *iChr-Notch-Mosaic* DNA construct inserted in the *Rosa26* locus. Below the genetic distance (kb) between different *LoxP* sites and the relative recombination ratios obtained after Cre-expressing plasmid transfection. (B) Representative picture of *iChr-Notch-Mosaic* ES cells expressing the different fluorescent proteins. (C) Confocal micrographs of 20-micron thick cryosections immunostained for the indicated markers. The border between the marginal and ventricular zones is indicated by a yellow line (see also Figure S3). (D) Dot plot of individual clones. Error bars indicate SEM. (E) Ratios of the recombined cells observed in ES cells and after differentiation to ECs. Below is shown a representative picture of an embryoid body (EB)-derived endothelial monolayer.

As an example of the utility of the method and these new genetic tools, we characterized the phenotype of genetic mosaics, in which cells can have normal, low, or high Notch or VEGF signaling. First, we evaluated the frequencies of recombination and eventual toxicity of the induced FPs in the *iMb-Control-Mosaic* and *iChr-Control-Mosaic* ES cell lines (Figures S2A and S2E). 2 days after the transient transfection of these control cell lines with Cre-expressing plasmids, we detected the different FPs in different proportions, and these were maintained after 8 days, showing that the expression of the different FPs *per se* is not toxic and does not alter the long-term cell proliferative behavior of cells (Figure S2I). As predicted from previous studies (Zheng et al., 2000), our results also confirm that in general there is an inverse correlation between the genetic distance separating the *LoxP* sites and the recombination frequency (Figures 2A and S2I). This suggests that it is better to place the shortest FP-gene cassettes in the first positions in order to have a more even chance of recombination among the three open reading frames (ORFs).

**Figure S3:**
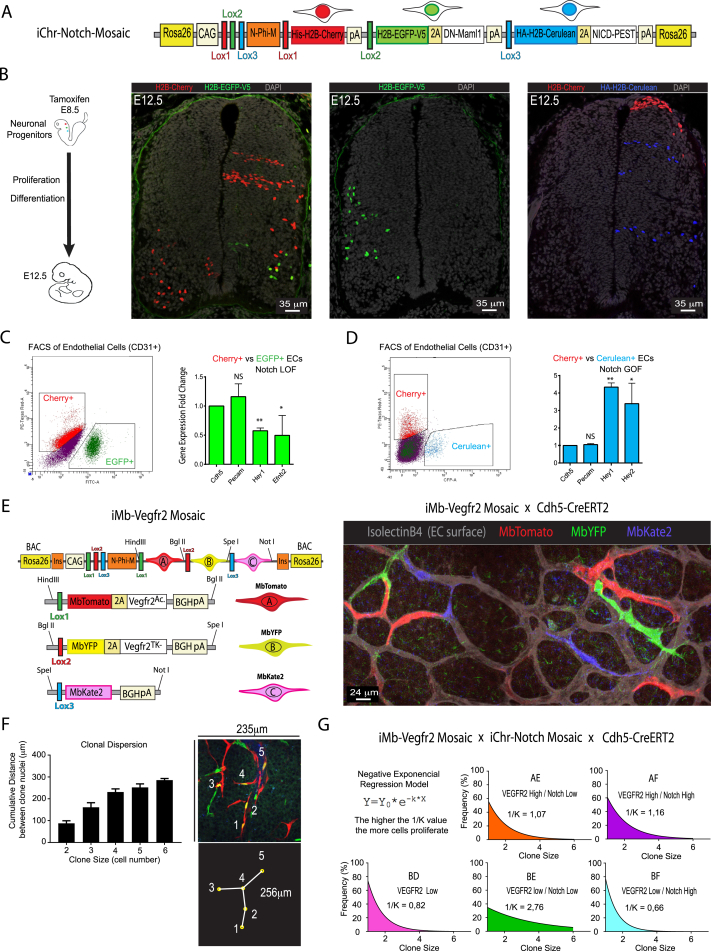
Inducible *iChr-Notch-Mosaic* and *iMb-Vegfr2-Mosaic*, Related to Figures 2–4 (A) Schematic representation of the genetic construct used to generate the *iChr-Notch-Mosaic* mice. (B) Representative pictures of *iChr-Notch-Mosaic Polr2a-*CreERT2 embryos with lower frequency of labelled clones in the neural tube, further supporting the data shown in Figure 2. (C and D) qRT-PCR analysis of RNA extracted from FACS sorted ECs from *iChr-Notch-Mosaic* mice. Canonical Notch signaling targets (Hey1, Hey2 and Efnb2) change significantly in cells with loss (GFP+ in [C]) or gain (Cerulean+ in [D]) of Notch function. NS, not significant, ^∗^ p<0.05 and ^∗∗^ p<0.001. *Cdh5* and *Pecam* are control endothelial genes not regulated by Notch. n=2, error bars represent Stdev. (E) Schematic representation of the genetic construct used to generate the *iMb-Vegfr2-Mosaic* mice. These mice were intercrossed with *Cdh5-CreERT2* mice and the progeny injected with tamoxifen at postnatal day 3 (P3). At P6, retinas were immunostained to detect the cellular mosaic as shown. (F) Chart showing the quantification of the average clone dispersion for each clone size. On the right a representative picture of a dual clone with 5 cells and a 256 micron cumulative distance between all nuclei. Error bars, s.e.m. This distance can be used to define areas for clone identification and quantification, in relation to their size. (G) Negative exponencial regression model used to derive the 1/K value, that gives an estimation of the proliferative capacity of the different cells shown in Figure 4F. BE cells are the most proliferative and the BF the least proliferative.

**Figure 3:**
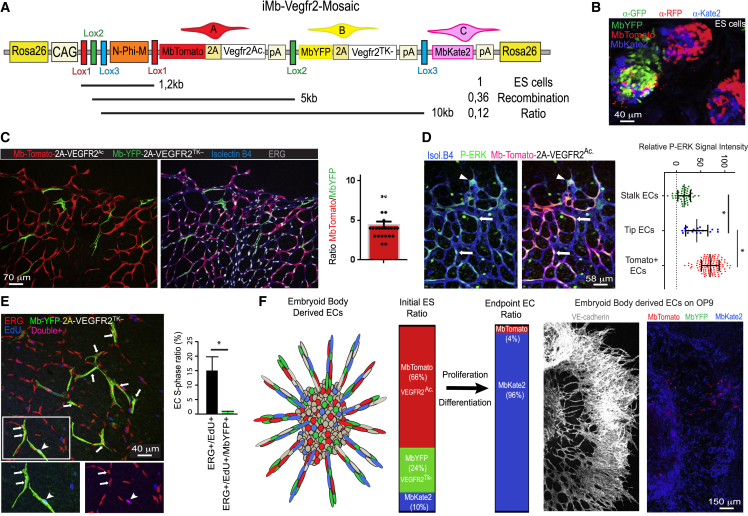
Inducible *iMb-Vegfr2-Mosaic* ES Cells and Mice (A) *iMb-Vegfr2-Mosaic* DNA construct. Below the genetic distance (kb) between different *LoxP* sites and the relative recombination ratios obtained after Cre transfection. (B) Representative picture of *iMb-Vegfr2-Mosaic* ES cells expressing the different fluorescent proteins. (C) Confocal micrographs showing the MbYFP- and MbTomato-expressing cells in a vasculature of a P6 mouse retina, 3 days after tamoxifen injection. IsolectinB4 labels the surface and ERG the nuclei of all ECs. Each dot in the chart indicates the identified ratio of EC surface area occupied by MbTomato- and MbYFP-expressing cells in each microscopic field. (D) Immunostaining of retinal vessels for phospho-ERK. Charts show that P-ERK signals are higher in most tip cells (arrowhead) and in MbTomato+ cells (arrows). (E) Immunostaining of retina ECs (nuclei, ERG+) for the indicated markers 3 days after the tamoxifen pulse. Most YFP+ cells have no EdU labeling (arrows) and few are EdU+ (pink nuclei, arrowhead inset). (F) Embryoid bodies derived from ES cells were plated on a OP9 monolayer to induce EC differentiation and sprouting. Colored bars indicate occurrence of each cell population among all recombined cells at the ES cell stage and after differentiation to ECs. In (D) and (E), error bars SD, ^∗^p < 0.05.

We next evaluated the functionality of these new *ifgMosaic* constructs in ES cells and mice. We studied the proliferation, migration, and differentiation of cells during neurogenesis and angiogenesis, processes known to be controlled by the Notch- and VEGF-signaling pathways. To induce the *iChr-Notch-Mosaic in vivo*, we crossed our mouse line with the *Polr2a-CreERT2* mouse line (Guerra et al., 2003), injected tamoxifen at embryonic day (E) 8.5, and collected embryos at E12.5 (Figure 2C). In the neural tube, single-progenitor cells proliferate and obey domain boundaries in the ventricular zone (VZ), forming stripes of neuronal progenitors (Briscoe and Small, 2015) that later differentiate and migrate to the mantle zone (MZ). The results obtained in *iChr-Notch-Mosaic* embryos indicate that single neuronal progenitors, pulsed with tamoxifen at E8.5 and having normal Notch levels (H2B-Cherry+), proliferate to give rise to more progenitors (Tuj1−, Sox2+), and at E12.5, only a subset of these had differentiated (Tuj1+, Sox2−) and were present in the MZ (Figures 2C, 2D, and S3B). This situation is optimal for sustaining neurogenesis for long periods of time and preventing progenitor exhaustion. Single-progenitor cells expressing *NICD-PEST* (H2B-Cerulean+), which increases Notch activity (Bray, 2006, Louvi and Artavanis-Tsakonas, 2006), differentiated poorly, and their progeny were found mainly in the VZ at E12.5. Interestingly, most of the progenitors expressing *DN-Maml1* (H2B-GFP-V5+), which decreases Notch activity (Maillard et al., 2004), form smaller clones because they commit to differentiation precociously and move laterally to the MZ, where terminally differentiated and non-proliferative cells accumulate (Figures 2C, 2D, and S3B). Sox2+ progenitor cells with higher Notch signaling also form smaller clones than the control/cherry+ cells, which proliferate and differentiate normally, suggesting that even though opposing Notch levels produce opposite cell-differentiation outcomes, they both result in impaired neurogenesis and reduced expansion of individual progenitor cells (Figure 2D). In addition to neurogenesis, we also evaluated *iChr-Notch-Mosaic* frequencies during endothelial cell (EC) differentiation from ES cells *in vitro*. Cells with low Notch signaling seem to have a competitive advantage in this assay, whereas ES cells with high Notch signaling cannot differentiate into ECs (Figures 2E, S3C, and S3D). We also used the *iMb-Vegfr2-Mosaic* mice to evaluate how ECs with different levels of VEGFR2 signaling behave during angiogenesis (Figure 3A). Cells expressing VEGFR2^Ac.^ (MbTomato+), which activates VEGFR2 signaling (Dosch and Ballmer-Hofer, 2010), have significantly higher levels of ERK phosphorylation, like endothelial-sprouting tip cells, and are frequently found at the vessel tips (Figures 3C and 3D). Cells expressing *Vegfr2*^*TK−*^ (MbYFP+), a tyrosine kinase mutant form (Y1173) of murine *Vegfr2* (Sakurai et al., 2005), which binds VEGF and dimerizes with wild-type VEGFR2, decreasing VEGFR2 signaling in a cell-autonomous manner, exit cell-cycle during angiogenesis (Figure 3E). ES cells expressing *Vegfr2*^*TK−*^ (MbYFP+) cannot differentiate to ECs, whereas some cells expressing VEGFR2^Ac.^ (MbTomato+) can differentiate but are outcompeted by the adjacent control (Kate2+) cells (Figure 3F), even though MbTomato+ cells have more VEGFR2 and ERK signaling. This surprising finding would be impossible to obtain using classical genetics.

### The Dual ifgMosaic Method Enables Multiple Mosaic Epistasis Analysis in the Same Tissue and at High Resolution

**Figure 4:**
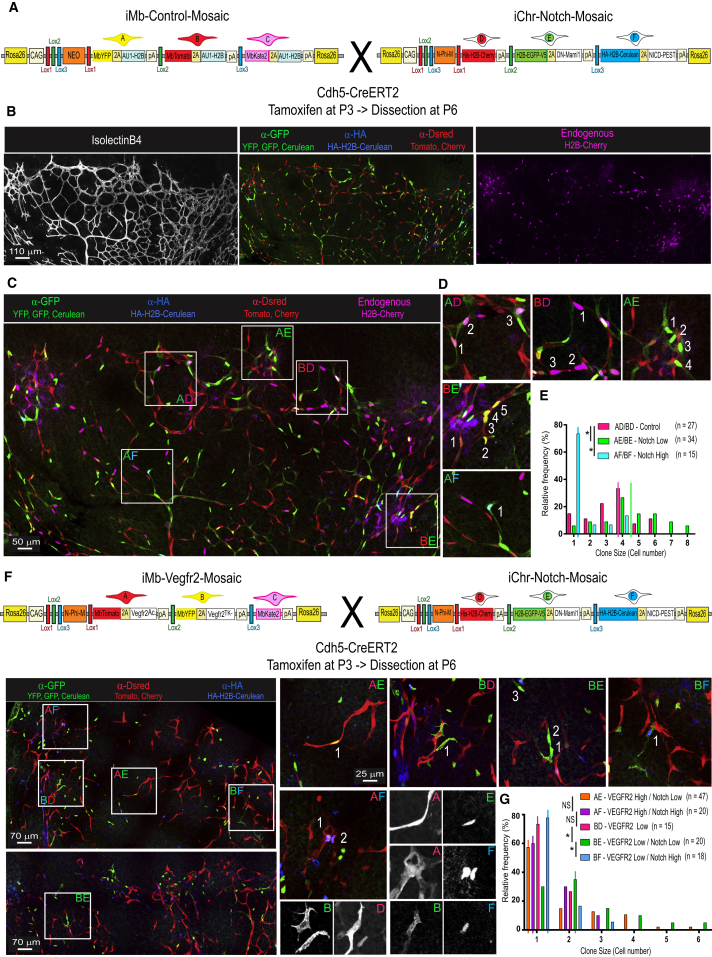
Single-Cell Resolution Epistasis Analysis with *Dual ifgMosaic* Mice (A) *iMb-Control-Mosaic* and *iChr-Notch-Mosaic* mice were intercrossed with *Cdh5-CreERT2* mice and progeny analyzed at P6. (B) Representative confocal micrographs of the retinas 3 days after tamoxifen induction, showing the five different acquisition channels and FPs obtained in *Dual ifgMosaic* mice. (C) Composite image of the pictures showed in (B), where is possible to see the different clones of cells. White boxed areas delimitate some Dual ifgMosaic clones. (D) Higher magnification pictures of the boxed areas (C) allow visualization of the cell shape and nuclei for quantification of clone size and distribution. Letter codes were assigned to double recombined clones according to their membrane (A–C) or nuclear (D–F) color. (E) Histogram showing the frequency and clone size of dual clones according to their nuclear color (Notch-signaling level). Vertical lines indicate median values. ^∗^p < 0.01. (F) Schematic representation of the genetic constructs of *iMb-Vegfr2-Mosaic* and *iChr-Notch-Mosaic* mice and representative confocal micrographs of retina vessels expressing the different combination of genes/FPs. On the right is shown higher-magnification pictures of some dual fluorescent clones. Isolated color channels of each dual clone are shown in gray. (G) Histogram showing the frequency and clone size according to their dual nuclei and membrane color (Notch- and VEGF-signaling level). Vertical lines indicate the median value for each type of clone. ^∗^ p < 0,05.

The results shown above highlight how genetic mosaics can be induced and used to study the role of genes in distinct cell populations. However, we very often need to understand the impact of genes not on groups of cells but rather on single cells and to understand their impact on single-cell heterogeneity. To accurately track single-cell proliferation and migration over time with the individual *iMb-Mosaic* or *iChr-Mosaic* mouse lines described above, recombination needs to be induced at relatively low frequencies to ensure single-cell or clonal resolution. However, this results in few clones of recombined cells per tissue, and it is therefore rare with this methodology to observe distinct clones in the same image-acquisition view, rendering mosaic analysis more difficult and time consuming. To obtain a higher number of clones and then track, with high accuracy, the proliferative and migratory behavior of different individual cells expressing different genes in the same tissue, we used the Dual ifgMosaic strategy. As a first example, we show the result of interbreeding the *iChr-Notch-Mosaic* and *iMb-Control-Mosaic* mouse lines (Figures 4A). Instead of inducing and analyzing only 3 distinct clones of cells, the Dual ifgMosaic strategy allows the generation of up to 15 distinct cell clones, as mentioned above (Figure 1), where only a subset of them have a dual color-code (Figures 4B–4D). The low frequency of dual recombination events makes it easier to identify, count the cell number, and define the shape of adjacent cell clones expressing up to three different genes in the same image-acquisition view, thus enabling a much more precise quantification of the effect of different genes on the proliferation, differentiation, and migration of individual cells over a pulse-chase period (Figures 4B–4D). With this strategy, we could accurately quantify how many times a single EC divides within a defined time window during angiogenesis (Figure 4E). We could also determine the average clonal-cell dispersion, according to its size (Figure S3F). To accurately quantify and delimit the most frequent dual clones in areas with higher recombination (Figure 4C), the average clone-dispersion value (Figure S3F) can be used to define an area (white squares in Figure 4C) that contains all cells of an individual clone.

With the Dual ifgMosaic strategy, it is also possible to perform multiple and combinatorial mosaic epistasis analysis with high temporal and cellular resolution, since it is possible to induce a mosaic of cells where the expression of up to three genes (A, B, or C) can be induced in combination with the expression of any other three genes (D, E, or F). The Dual ifgMosaic method therefore enables epistasis analysis of up to six genes or genetic pathways in up to nine different expression combinations, at single-cell resolution and in the same tissue. As an example, we crossed the *iChr-Notch-Mosaic* and *iMb-Vegfr2-Mosaic* mice to analyze how single cells with different combinations of Notch and VEGF signaling proliferated over time when confronting the same biological context (Figures 4F, 4G, and S3G). This analysis revealed that activation of VEGFR2 signaling in high-Notch cells does not significantly increase their proliferation (compare bars AF in Figure 4G with 4E) and that single ECs with low VEGF signaling, which generally do not proliferate, can divide and generate larger clones when Notch signaling is low but not when Notch signaling is high (BD vs BE vs BF in Figure 4G). Surprisingly, we also found that a large fraction of single ECs with simultaneous high VEGF and low Notch signaling, which are both pro-mitogenic stimuli, do not proliferate, whereas most single control or low-Notch cells proliferate well (compare AE in Figure 4G with AE/BE or AD/BD in 4E, and see also S3G).

### PB-Rosa26 BAC for Easy Recombineering of the ifgMosaic and Robust Single-Copy Transgenesis

**Figure S4:**
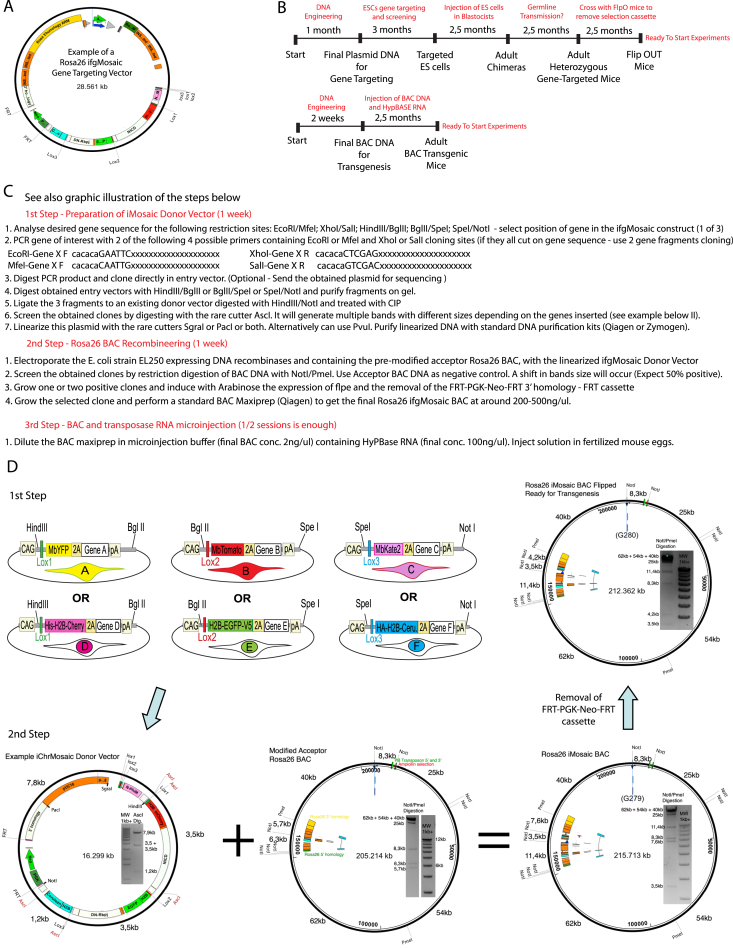
Overcoming Limitations of Classical Gene Targeting through the Generation of *Rosa26 ifgMosaic* BACs, Related to Figure 5 (A) Map of a typical *Rosa26* gene-targeting vector containing 28.5kb of DNA encoding all the elements required for precise *Rosa26* gene targeting and strong Cre-dependent induction of a fluorescent-genetic mosaic. These vectors are very difficult to assemble due to their large size and multiple repetitive elements. (B) Comparison of the time required to generate an *ifgMosaic* mouse line by classical gene targeting versus the new *Rosa26 ifgMosaic* BAC method. (C) Three step method for the engineering of *Rosa26 ifgMosaic* BACs. (D) Plasmids and BACs used at the different steps to generate the final *Rosa26 ifgMosaic* BAC. Restriction analysis gel pictures indicate the successful engineering of the DNA constructs at the different steps.

The induction of functional genetic mosaics with the *ifgMosaic* strategy requires the genome integration of a large multi-ORF construct as a single copy. We initially achieved this by gene targeting of the safe-harbor *Rosa26* locus in mouse ES cells (Soriano, 1999). However, we encountered difficulties in cloning these large plasmids, which can be as large as 30 kb (Figure S4A) and contain multiple genes and repetitive sequences, including the four insulators, six *loxP* sites, four transcription stop sequences, and FPs with very similar sequences (YFP, GFP, and Cerulean). We also detected frequent stochastic deletions during plasmid amplification. Assembling all these elements in a single construct and screening out these sporadic deletions was time consuming and made generation of the desired large *ifgMosaic* constructs, with all the required elements for gene targeting and induction, difficult and unpredictable. Once these problems were overcome, we also had difficulties obtaining mouse ES cell clones with correct targeting of the *Rosa26* locus. This is likely due to the large size of the *ifgMosaic* constructs and the existence of multiple direct repeats. Using traditional gene-targeting technology, inserts of up to 5 kb can be targeted to the *Rosa26* locus in mouse ES cells at a typical targeting efficiency of 15%; but with DNA inserts of around 25 kb containing multiple repeats, only 2% of clones were correctly targeted (detected by PCR or Southern blot).

**Figure 5:**
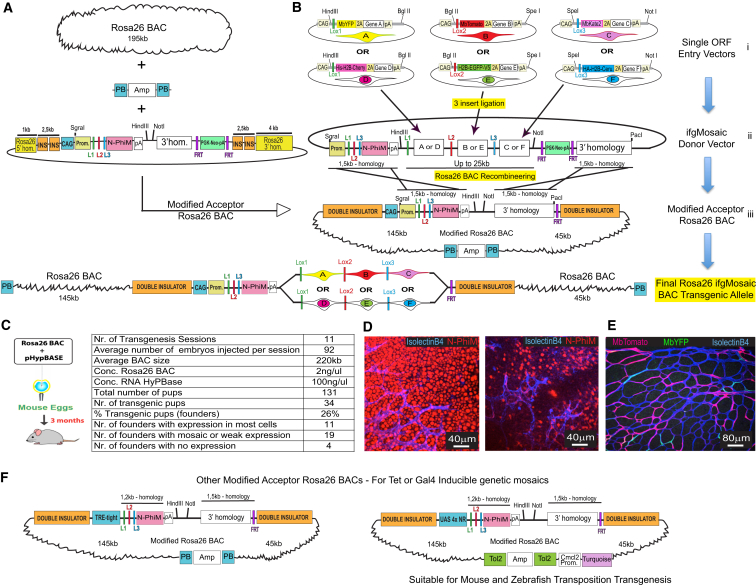
*Rosa26 ifgMosaic* BAC Recombineering and Transgenesis (A) A BAC containing 195 kb of the euchromatic *Rosa26* locus was first modified to include the piggyBac (PB) transposon elements flanked by the ampicillin (Amp) selection cassette and all the other elements necessary for robust BAC recombineering, transgenesis, and expression. (B) Three-step pipeline for generating *Rosa26 ifgMosaic* BACs. (1) The desired genes (A–F) are cloned in frame downstream of the 2A-peptide sequences of each construct. (2) Three individual DNA fragments (HindIII-BglII, BglII-SpeI, and SpeI-NotI) are obtained from the entry vectors and ligated simultaneously to an existing acceptor vector (digested with HindIII/NotI) to generate the *ifgMosaic* donor vector. (3) This *ifgMosaic* donor vector includes two 1.5 kb homology arms for precise and efficient BAC recombineering in *E. coli*, which allows the generation of the *Rosa26 ifgMosaic* BAC. This BAC is then flipped in *E. coli* to remove the *FRT-PGK-Neo-FRT* cassette and subsequently used for piggyBac-transposon-mediated transgenesis in mouse zygotes. (C) Efficiency of the *Rosa26 ifgMosaic* BAC transgenesis method. (D) Expression of the N-PhiM reporter indicates activity of the BAC *Rosa26-INS-CAG* promoter in transgenic lines with ubiquitous and variegated expression. (E) Representative confocal micrograph of the retina vasculature of mice containing the BAC *Rosa26* transgenic allele after tamoxifen induction of *Cdh5-CreERT2*, which results in recombination and expression of the mosaic in ECs (IsolectinB4+). (F) Additional modified acceptor *Rosa26* BACs for Tre/Tet or UAS/Gal4 induction.

To overcome these problems, we devised new cloning strategies to facilitate the assembly of the desired large multi-ORF DNA constructs and a method that would also yield mice ready for experimental breeding in only 3 months, instead of the typical 12 months required with classical gene targeting (Figure S4B). Our method is based on the injection of an engineered *Rosa26* BAC (bacterial artificial chromosome) together with transposase RNA into mouse eggs (Figure 5A–5C). Compared with plasmids, BACs are able to carry and replicate much larger quantities of DNA (around 200–250 kb). We first modified an existing BAC that includes 200 kb of the euchromatic *Rosa26* locus, which is known to be actively expressed in all mouse cells. By DNA recombineering in bacteria, we inserted several sequences that together allowed us to generate robust transgenic and unicopy *ifgMosaic* mice. The inserted sequences were as follows: the piggyBac (PB) transposon inverted repeats, for efficient single copy and complete BAC integration in the genome; four insulator (INS) sequences flanking the required transcriptional units, to decrease the probability of regulatory interference from neighboring genomic regions; the strong and ubiquituous CAG enhancer and promoter, to enhance gene expression; 3 kb of DNA homology sequences, to promote efficient BAC-plasmid DNA intermolecular recombineering in bacteria; and the N-PhiM reporter of CAG promoter expression in the absence of Cre activity (Figure 5A). N-PhiM is a noncytotoxic modified PhiYFP gene (Evrogen) with a nuclear localization signal and lacking endogenous fluorescence (Cai et al., 2013), and it can be detected by immunostaining (Figure 5D). The inclusion of the large 3 kb homology sequences was necessary to outcompete intramolecular recombination between the several DNA repeats present in these multi-ORF mosaic constructs. Recombineering the engineered *Rosa26* BAC in *E. coli* supported highly efficient in-frame insertion of large *ifgMosaic* DNA fragments (up to 25 kb) containing up to four large transcriptional units (Figure 5B). We provide step-by-step guides for the rapid assembly of the donor multi-ORF constructs, BAC recombineering, and screening for the full integration of these constructs in the acceptor *Rosa26* BAC through restriction enzyme analysis and simple gel electrophoresis (Figures S4C and S4D). Overall, this method significantly simplifies the DNA cloning steps because the donor vectors for BAC recombineering are 12 kb smaller than the final classical gene-targeting vectors used to target the *Rosa26* locus (compare Figures S4A and S4D).

**Figure S5:**
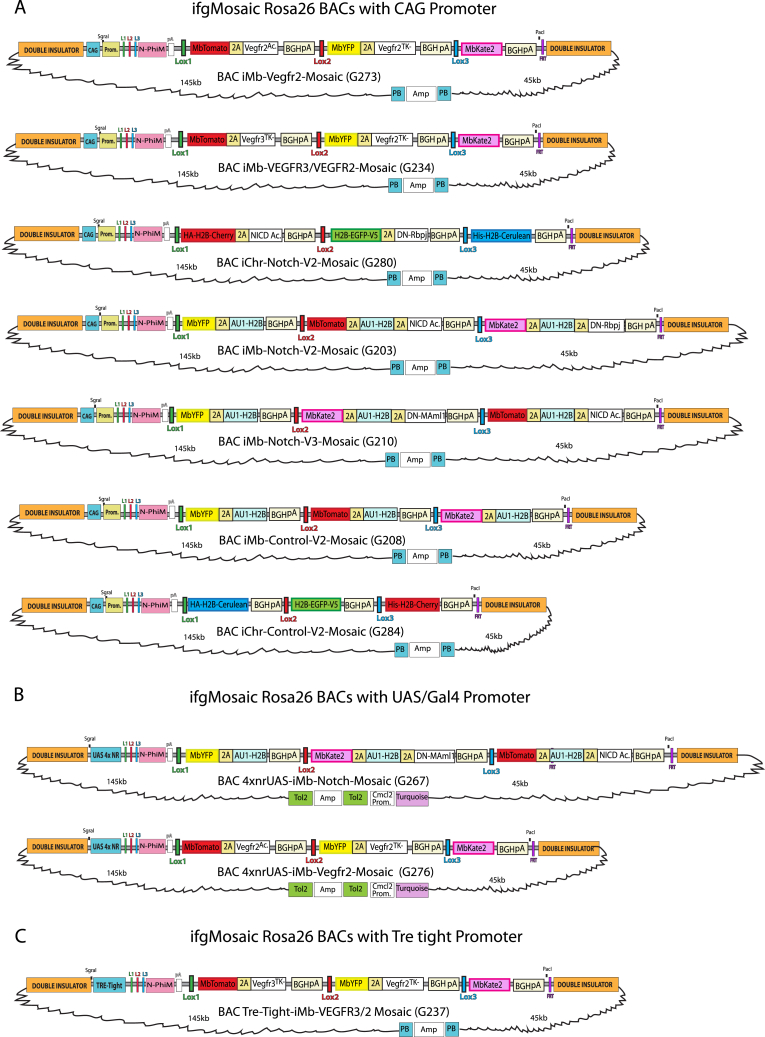
*Rosa26 ifgMosaic* BACs, Related to Figure 5 (A–C) Maps of selected *Rosa26 ifgMosaic* BACs generated by DNA recombineering. These BACs contain the CAG (A), UAS 4x NR (B), or Tre-Tight (C) promoters.

Having obtained the desired modified *Rosa26 ifgMosaic* BAC (Figure S5A), we injected it together with the RNA encoding the hyperactive piggyBac transposase (HyPBase) into fertilized mouse eggs (Rostovskaya et al., 2013). The piggyBac transposition system allowed us to obtain on average 26% BAC-transgenic pups with complete genomic integration of a single BAC; in contrast, standard methods of BAC linearization, purification, and injection yielded only 3% transgenic offspring. The transposase-dependent transgenesis method yielded on average three founder animals from a single transgenesis session, representing significant time and cost savings. Screening of F1 transgenic mice containing these modified *Rosa26 ifgMosaic* BACs revealed N-PhiM reporter expression in most cells of 32% of founders (Figures 5C and 5D), and reporter expression was maintained across all generations examined, as with classical *Rosa26* locus gene targeting. The frequency of *LoxP* recombination and the expression level of these transgenic *Rosa26* BACs are also identical to those observed with classical *Rosa26* gene targeting (Figure 5E). We also generated acceptor and *Rosa26* BAC constructs containing the Tre-tight and upstream activating sequence (UAS) 4xnr promoters, which allow titratable and reversible expression of the mosaic in cells expressing tTA/rtTA or Gal4, respectively (Figures 5F, S5B, and S5C). With these DNA constructs, any laboratory can easily generate *ifgMosaic* cell lines, zebrafish, or mice. In addition, since the *Rosa26* BAC integrates in the genome randomly and not in the *Rosa26* locus, the mouse lines can be crossed with other available mouse lines containing other classical *Rosa26* knockin alleles. In summary, this method of BAC recombineering and transgenesis allows the rapid and robust generation of single-copy transgenic cells or animals ready for inducible mosaic gene-function analysis.

### CRISPR/Cas9-Mediated Integration of Large ifgMosaic Constructs in a Pre-modified Rosa26 Locus in mES Cells

**Figure 6:**
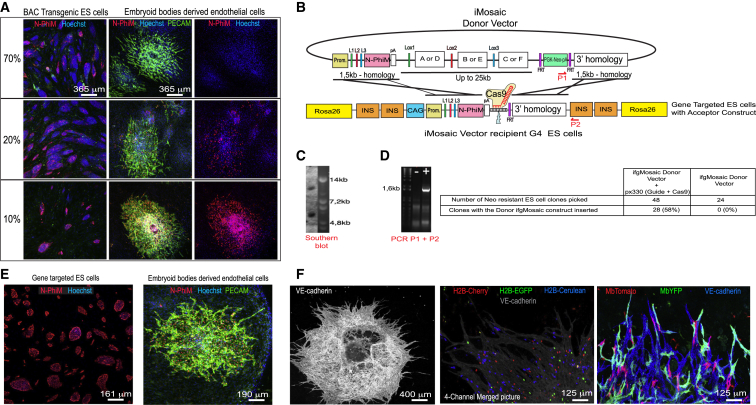
*Rosa26 ifgMosaic* Gene Targeting in ES Cells (A) Confocal micrographs showing expression of the N-PhiM reporter in BAC transgenic ES cells and differentiated ECs (Pecam+). All neomycin-resistant clones show expression of the BAC in ES cells, but only 10% have robust expression in virtually all ES and ECs. (B) Cas9-mediated gene targeting of a donor *ifgMosaic* vector into the endogenous *Rosa26* locus, premodified to contain an acceptor construct with 3 kb of homologous sequences and a Cas9 target sequence for highly efficient and precise gene targeting by homology-dependent double-strand break repair. (C) Southern blot analysis of gene-targeted ES cell clones containing the acceptor construct in the *Rosa26* locus. (D) PCR screening reveals that 58% of neomycin-resistant ES cell clones have the desired integration of the *ifgMosaic* cassette into the premodified *Rosa26* locus. (E) Expression of the N-PhiM reporter in gene-targeted ES cells and ECs (Pecam+). (F) Induction of recombination *in vitro* in embryoid-body-derived ECs, showing mosaic expression of the three H2B- or Mb-tagged FPs in different ECs (VE-cadherin+).

Inducible genetic-fluorescent mosaics are also very useful tools for studying the biology of stem cells and their differentiated progeny *in vitro*. For example, the distribution of mosaic cell ratios among the progeny of specific progenitor cells can reveal how specific genes control the differentiation of certain cell types. We transfected mouse embryonic stem (mES) cells with the same modified BACs and hyperactive transposase plasmid and used neomycin and qRT-PCR to select clones containing a single copy of the genome-integrated BAC. Even though 95% of transgenic mES cell clones expressed the N-PhiM reporter, only 30% of them maintained reporter expression after differentiation to ECs, and only 10% had expression in most ES cells and ECs (Figure 6A). This result contrasts with the *in vivo* findings, which showed ubiquitous and homogenous reporter expression in 32% of the founders (Figures 5C and 5D). The main difference between the *in vivo* and *in vitro* BAC transgenesis approaches is the use in stem cells of the PGK-Neo selection marker to select the rare genomic integration events. This cassette has been shown to induce upstream promoter silencing (Ema et al., 2006). For the *in vivo* procedure, this cassette is removed from all BACs before egg microinjection and transgenesis. Removal of the *FRT-PGK-Neo-pA-FRT* cassette from BACs integrated in ES cells, by *Flpe*-encoding plasmid transfection, led to an increased frequency of N-PhiM reporter expression in only 25% of the transgenic clones (data not shown), at the cost of doubling the time required to generate *ifgMosaic* ES cells.

Given the weak improvement in BAC expression *in vitro*, we explored strategies for direct gene targeting of the endogenous *Rosa26* locus in mouse ES cells. Classical gene targeting of the large *ifgMosaic* constructs is very inefficient, with correct gene targeting in only 2% of neomycin-resistant ES cell clones, probably due to the large size and the presence of multiple direct repeats in *ifgMosaic* constructs. We therefore decided to test a new method of *Rosa26* gene targeting—potentially more efficient and compatible with the smaller donor multiple-ORF *ifgMosaic* constructs that were used to recombine the *Rosa26* BACs in bacteria (Figures 6B and 5B). Unlike classical *Rosa26* gene-targeting constructs (Figure S4A), these are much easier to construct because they lack the large homology arms (required for classical gene targeting), the CAG promoter and the insulator sequences, making them 12 kb shorter. We hypothesized that the CRISPR/Cas system would significantly enhance homologous recombination (HR) and the efficiency of gene targeting in a pre-modified *Rosa26* locus containing all the constant elements present in all the *ifgMosaic* constructs. To test this, we first generated an acceptor mES cell line containing in the *Rosa26* locus the elements previously inserted in the acceptor *Rosa26* BAC (compare Figures 5A and 6B). In the center of this cassette, we included a unique sequence to enable highly efficient and guided CRISPR/Cas9-mediated DNA double-strand breakage (Figure 6B). Nucleofection of the new acceptor G4 mES cells, with the previously generated donor multi-ORF *ifgMosaic* constructs and the px330 plasmid encoding Cas9 and the unique single-guide RNA (sgRNA) sequence, allowed us to induce double-strand breaks at the desired integration site. After 7 days of selection with neomycin, we picked clones from each *ifgMosaic* donor plasmid nucleofection, finding that 58% of ES cell clones had integration of the desired donor construct by homologous recombination in the pre-modified *Rosa26* locus (Figures 6C and 6D). This method increased the efficiency of correct *Rosa26* gene-targeting of multi-ORF *ifgMosaic* constructs by 30-fold compared with classical gene targeting using large constructs (2%). Importantly, and in contrast with the *in vitro* BAC transgenesis method, all the *Rosa26*-targeted clones had high expression of the N-PhiM reporter in most stem cells and differentiated progeny (Figure 6E). In addition, we could induce the expected cellular mosaic in ES cells and differentiated progeny (Figure 6F). Therefore, this novel acceptor ES cell line and the method provided here greatly simplify the generation and targeting of multi-ORF *ifgMosaic* constructs to the *Rosa26* locus and can be used for the high-throughput generation of multiple *ifgMosaic* ES cell lines for *in vitro*-inducible genetic-fluorescent mosaic analysis. The generated acceptor ES cells are from the G4 background and have a very high germline-transmission potential (George et al., 2007). We successfully used them to generate gene-targeted *Rosa26 ifgMosaic* mouse lines.

### Design and Testing of Improved Versions of the ifgMosaic Constructs

**Figure 7:**
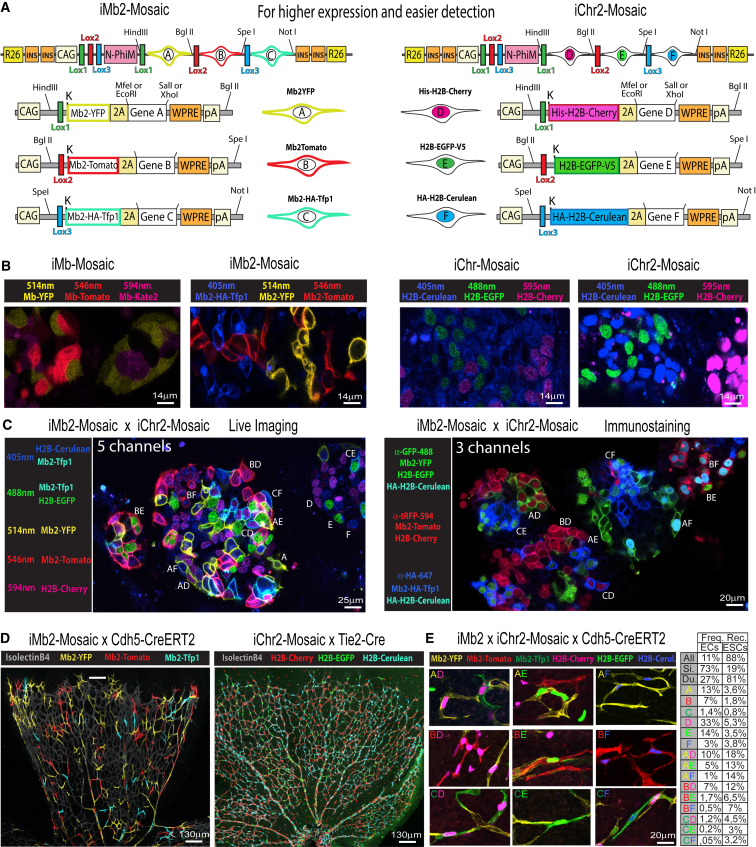
Second-Generation Improved *iMb2-Mosaic* and *iChr2-Mosaic* Constructs and Cell Lines (A) Schematic illustration of the *iMb2-Mosaic* and *iChr2-Mosaic* DNA constructs (see also Figure S7). (B) Live imaging and comparison of endogenous fluorescence intensity in the *ifgMosaic* and the improved i*fg2Mosaic* ES cell lines. (C) Combinatorial Dual ifg2Mosaic endogenous fluorescence in ES cellular genetic mosaics after live scanning confocal microscopy or after immunostaning with the indicated antibodies. Two-letter codes indicate the double-recombined cell clones and the observed combination of FPs A–F, confirming that it is possible to distinguish all the cell clones in the same acquisition field and with the same microscopy settings for direct cell comparison. (D) Expression of *iMb2-Mosaic* and *iChr2-Mosaic* in P6 mouse retina ECs using the inducible *Cdh5-CreERT2* or *Tie2-Cre* mouse lines. (E) Selected dual-labeled clones of retina vessels pulsed with tamoxifen once (see also Figure S6). Numbers indicate the frequencies of recombination and labeling obtained in Cdh5-CreERT2+ ECs, pulsed once with tamoxifen, or in embryonic stem cells (ESCs) transfected with Cre- and Puro^R^-expressing plasmids and grown on Puro for 3 days. “All” indicates the frequency of all recombined/fluorescent cells in the tissue. The relative single (Si.) and dual (Du.) recombination frequencies change according to the level of Cre activity, which is higher in transfected ESCs.

**Figure S6:**
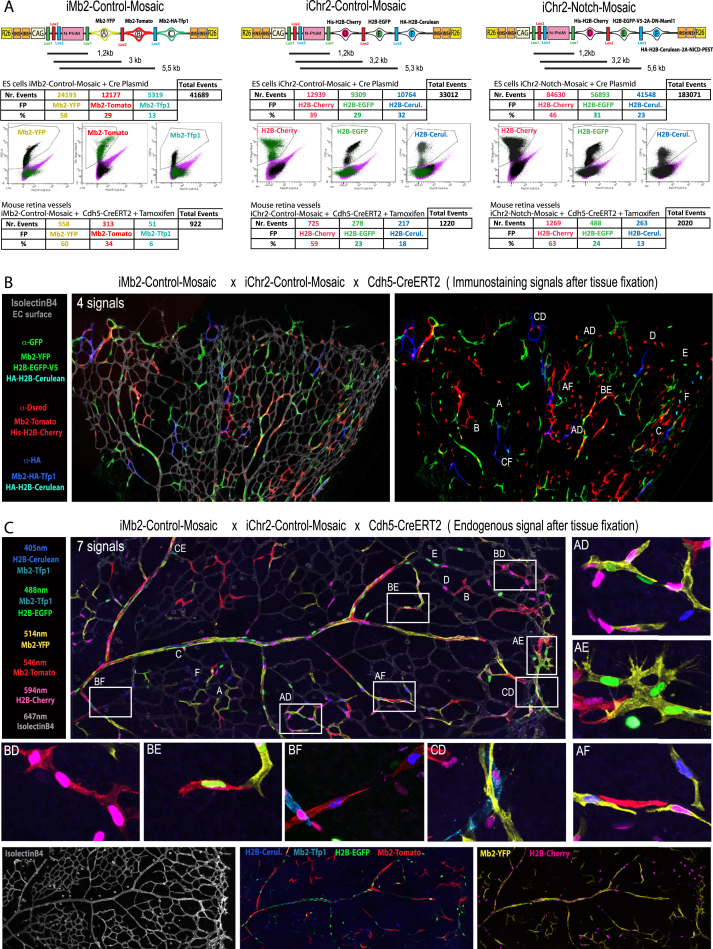
*iMb2-* and *iChr2-Mosaic* Recombination Frequencies and Signals, Related to Figure 7 (A) Diagrams showing the genetic distances between the *LoxP* sites in the DNA constructs used to produce the different *ifg2Mosaic* mice. Genetic distance is not the only factor that influences the relative ratios of recombination. The frequencies (%) indicated above the FACS plots were obtained in ES cells transfected with Cre-expressing plasmids and analysed by FACS, and later confirmed also by immunostaining. The frequencies below were obtained by confocal microscopy of ECs from *ifg2Mosaic Cdh5-CreERT2*+ mice, pulsed once with tamoxifen. The differences observed in the recombination ratios are caused by the higher levels of Cre activity obtained in ES cells than in ECs expressing CreERT2 and pulsed only once with tamoxifen. (B) Confocal micrograph showing the 4 signals detected after immunostaining with the indicated antibodies, of the postnatal mouse retina of *Dual ifg2Mosaic* mice, carrying the *Cdh5-CreERT2* allele, and pulsed once with tamoxifen. (C) Endogenous fluorescence detection in endothelial cells of the same mice after retina tissue fixation, and labeling with conjugated IsolectinB4-647. A total of 7 fluorescent signals (6 endogenous and 1 immunolabeled), excited with the indicated laser lines, could be detected with high resolution and in a large acquisition field. Magnified boxed areas and two-letter codes show selected double-recombined cell clones and the observed combination of FPs A to F, at higher magnification. Pictures further below show the different signals separately.

**Figure S7:**
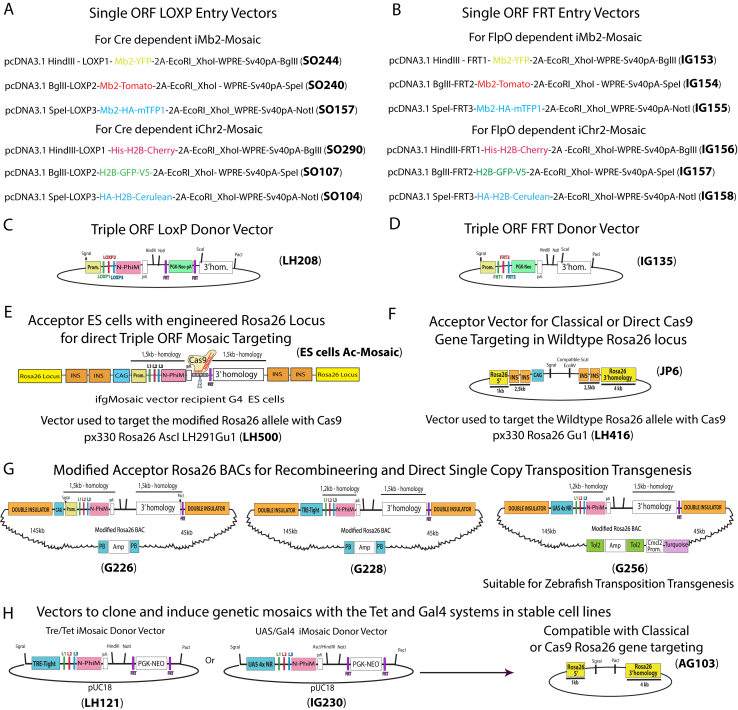
List of All DNA Constructs Used to Produce the Different Second-Generation *ifgMosaic* Constructs, Related to Figures 5–7 (A and B) *LoxP*- (A) or *FRT*-containing (B) entry vectors used to clone the desired genes in frame with the upstream FPs and the 2A peptide. (C and D) Map of *LoxP*- (C) or *FRT*-containing (D) donor vector used to clone the 3 cassettes from (A) or (B) in the HindIII/NotI sites. (E and F) The Triple ORF donor vectors of C and D can be digested with SgraI/PacI or SgraI/ScaI to insert the mosaic by Cas9 recombineering in pre-modified ES cells (E) or by ligation to *Rosa26* gene targeting vectors (F). Plasmids LH500 or LH416 are required to express and guide the Cas9 to the pre-modified (E) or wildtype (F) *Rosa26* locus. (G) The large SgraI/PacI fragments generated in C and D can be inserted by recombineering in different acceptor *Rosa26* BACs containing the following promoters: CAG, Tre-Tight, or UAS 4x NR (4 UAS elements non-repeated). BAC G256 can be used for transgenesis in zebrafish and contains a marker to directly select transgenic founders (Cmcl2-turquoise) based on turquoise fluorescence in the heart. (H) Smaller vectors that can be used to directly clone the mosaic constructs downstream of the Tre-Tight or UAS promoters for titratable and reversible induction. These vectors can also be digested with the rare cutters SgraI/PacI and cloned in a plasmid (AG103) containing the *Rosa26* homology arms for gene targeting. LOXP1, *LoxN*; LOXP2, *Lox2272*; LOXP3, *LoxP*; FRT1, *F3*; FRT2, *5T2*; FRT3, *545*; 2A, viral peptide allowing equimolar expression of multiple independent proteins from a single ORF; Mb2, second generation membrane tag; HA, V5 and His (small epitopes that can be used for specific antibody detection); H2B, histone tag that targets proteins to the chromatin/nucleus; WPRE, Woodchuck Hepatitis Virus Posttranscriptional Regulatory Element that enhances gene expression; Sv40pA, polyadenylation signal to stop transcription; N-PhiM, non-fluorescent protein that is used as a reporter of promoter expression; CAG, Strong and ubiquitous promoter; PGK-Neo, resistance marker for ES cell selection; INS-INS, Double Chicken B-globin insulator to increase gene expression and minimize regulatory interference.

The experience gained from the analysis of the previous *ifgMosaic* ES cell lines and mice prompted us to develop improved constructs (Figures 7A and S6A). These second-generation constructs (*iMb2-Mosaic* and *iChr2-Mosaic*) significantly enhance the expression of the FPs and downstream functional genes (Figure 7B), which is important for high resolution and nontoxic live imaging (Movie S2), and for better mosaic gene gain and loss-of-function function analysis. In addition, the combination of proteins and selected epitopes further facilitates the detection of the Dual ifgMosaic by live imaging or after immunostaining (Figures 7C, 7D, S6B, and S6C), which is essential for high-throughput mosaic quantitative analysis. During the development of *iMb2-Mosaic* and *iChr2-Mosaic* constructs, we overcame some of the limitations of previous *ifgMosaic* constructs. First, when the ORF coding the fluorescent protein and the gene of interest is large and contains one or two 2A peptide sequences, expression of the functional genes and FPs is relatively low due to the reported 2A-peptide-induced translation pause and ribosomal skipping step (Sharma et al., 2012, Trichas et al., 2008). We overcame this problem by including several genetic elements that overall significantly increased the expression of the *ifgMosaic* ORFs. For instance, we introduced in all ORFs the full consensus KOZAK sequence (gccaccATGgcg) (Kozak, 1987), and the WPRE element (Lee et al., 2005), which increases protein levels by increasing mRNA translation and stability (Figures 7A and 7B). Second, we included four insulators flanking the transcriptional units, for higher expression of the CAG promoter. Third, the membrane tag in the first-generation *ifgMosaic* constructs was substituted with an improved membrane tag (De Paola et al., 2003) to increase signal intensity at the membrane and decrease the signal in the cytoplasm (Figure 7B). This is especially important when an *iMb-Mosaic* is combined with an *iChr-Mosaic*, in which the separation of nuclei and membrane signals is crucial (Figure 7C, 7E, and S6C). And fourth, we noticed that the endogenous fluorescence of some proteins, such as Kate2, is reduced when they contain tags that target them to the membrane or nucleus or when they contain the 2A peptide in the C-terminal position (Figure 1E). To circumvent this problem, we used instead Mb2-HA-Tfp1-2A, which is a brighter blue-green (teal) FP and more compatible with the other FPs in the Dual ifg2Mosaic system (Figures 7B–7E and S6). We next analyzed the frequency of recombination obtained in *Dual ifg2Mosaic* ES cells transfected with Cre-expressing plasmids and in mice carrying the *Cdh5-CreERT2* allele and pulsed once with tamoxifen. This analysis revealed that the relative recombination ratios depend on the level of Cre expression or induction, the genetic distance between *LoxP* sites, and the nature of the DNA sequence of the mosaic construct used (Figure 7E and S6A). The higher the Cre activity, the more similar the recombination among the different *LoxP*-flanked ORFs and the higher the frequency of dual-labeled clones (Figure 7E). Overall, the relatively low frequency of each dual and combinatorial recombination event enables higher resolution clonal analysis, both in situations of low or high Cre activity. Of note, the genetic distance between *LoxP* sites is an important factor, but recombination is also influenced by the adjacent genes and FPs DNA sequences, since *iMb2-Control-Mosaic*, *iChr2-Control-Mosaic*, and *iChr2-Notch-Mosaic* ES cells and mice have significantly different recombination patterns despite very similar inter-*LoxP* distances (Figure S6A). We also cloned *FRT* sites in iMb2 and iChr2 mosaic constructs (Figures S7B and S7D). The *FRT*-containing *ifg2Mosaics* can be induced by Flpo or Flpo-ERT2 (Kranz et al., 2010, Lao et al., 2012). This expands the flexibility of the method. As an example, when a Cre-dependent *iMb2-Mosaic* allele is combined with an FlpO-dependent *iChr2-Mosaic* allele, *Dual ifgMosaics* can be induced that are dependent on two distinct recombination events that can occur sequentially over time, in the same or in distinct cells. This enables higher resolution cell-fate mapping or the study of sequential mosaic gene function and epistasis, which can be used to define how two genes sequentially modulate a given cell-lineage specification.

## Discussion

Previous DNA constructs and mouse lines based on the Brainbow technology (Livet et al., 2007, Snippert et al., 2010) enabled the generation of fluorescent cellular mosaics but not genetic functional mosaics. The first requires the assembly of up to four relatively small cassettes encoding the desired FPs downstream of different and mutually exclusive *LoxP* sites. To generate constructs for fluorescent and genetic functional mosaics, the cloning strategy has to be significantly different due to the larger size of the cloned genes and the required restriction-site versatility and compatibility at each cloning step. We therefore decided to implement new cloning strategies and design novel constructs that could be easily used by any scientist to insert genes of interest downstream of the FPs (Figures S2 and S7). This greatly facilitates the assembly of the final *ifgMosaic* constructs for direct transgenesis or gene targeting. The use of the viral 2A peptide allows the level and type of FP expression to be correlated with 100% certainty to the expression level of the downstream gene of interest. With the Brainbow system, it is difficult to detect complex fluorescent mosaics simply by scanning of the endogenous signal after combinatorial and stochastic recombination of multiple Brainbow constructs, especially if the cells are not clearly spatially separated or when the endogenous fluorescent signals are weak due to intrinsic low expression or tissue fixation. With the *Dual ifgMosaic* strategy, the complexity of the fluorescent mosaic arises from the recombination not of multi-copy transgenes but of just two *Rosa26* gene-targeted or two *Rosa26* BAC transgenic unicopy alleles (*iMb-Mosaic* and *iChr-Mosaic*). In addition, distinction among the 15 recombination possibilities is based on the detection of FPs that differ not only in their spectral profiles but also in their epitopes and cellular localizations, allowing simultaneous tracking of cell shape (membrane-localized FPs) and number (nuclear-localized FPs) of each dual-recombined cell clone by live imaging or immunostaining.

To achieve this, we had to investigate the different spectral properties and *in vivo* toxicity of all available FPs (Cai et al., 2013), identify which small-epitope tags could be used and easily detected *in vivo*, and determine the availability of specific and compatible antibodies (see STAR Methods). The experience gained led us to develop a second generation of functional *ifgMosaic* constructs and mouse lines (*iMb2-Mosaic* and *iChr2-Mosaic*) that not only enhances the expression of the FPs and downstream functional genes but also facilitates simultaneous detection of the several marker proteins in the same tissue with only three laser channels or antibodies. This is critical because most microscopes only allow detection of four to six different signal combinations. Therefore, with the *Dual ifgMosaic* strategy, the functional and complex genetic mosaics can be detected in the context of other stained tissue markers, which is very important for accurate localization of the different color-coded clones within the tissue and quantitative phenotypic analysis. This strategy not only supports multiplex dual labeling of the membrane and chromatin in recombined cells for higher-resolution analysis of the clone-cell number and shape but also allows multiple and combinatorial genetic-epistasis analysis at single-cell or clonal resolution, because the function of three genes can be studied in the context of any other three expressed genes.

We also present two methods that greatly simplify the generation of transgenic and gene-targeted *ifgMosaic* ES cell lines and mice. The first method is based on *Rosa26* BAC recombineering and requires only standard DNA-cloning techniques and a standard transgenesis service. This fast method generates adult transgenic *ifgMosaic* animals, ready for breeding and experiments, just 3 months after starting the DNA construct cloning and requires only 2 weeks of hands-on time. With the second method, Cas9-induced HDR (homologous-dependent recombination) between the *ifgMosaic* donor vector and the pre-modified *Rosa26* allele occurs in more than half of the Neo-resistant ES cell clones, allowing multiple ES cell lines to be generated with different *ifgMosaic* constructs in only 2 weeks. The phenotype of these stem cells or their differentiated progeny can be directly studied by high-throughput live imaging (Movie S2) or FACS sorting (Figure S6A). The insulated *Rosa26-CAG* promoter used is strong and ubiquitous (Muzumdar et al., 2007, Soriano, 1999), allowing induction of the *ifgMosaic* in any cell type. To provide higher versatility of mosaic induction in any cell or model organism, we also assembled *ifgMosaic* constructs with UAS- and Tre-tight-inducible promoters and FlpO-inducible *FRT* elements.

In summary, these new DNA constructs (Figure S7), mice, and methods will significantly ease high-resolution mosaic gene-function analysis, which is crucial for distinguishing between cell-autonomous and non-cell-autonomous gene functions. The *Dual ifgMosaic* approach will additionally allow investigation of how up to six genes or genetic pathways interact functionally at the single-cell level when they are expressed in different combinations in cells facing the same environment. Since all the analysis with this approach can be done in the same animal, tissue, or image acquisition field, phenotypic and statistical comparisons between different mutant and adjacent control cells will be more insightful than those made with current standard gene-function-analysis approaches, which require comparison between cells present in separate control and mutant animals.

## STAR★Methods

### Key Resources Table

**Table:** 

REAGENT or RESOURCE	SOURCE	IDENTIFIER
**Antibodies**		

Rabbit Anti-GFP	Clontech	Cat# 632592; RRID: AB_2336883
Goat Anti-GFP	Acris Antibodies	Cat# R1091P; RRID: AB_1002036
Rabbit anti-Dsred	Clontech	Cat# 632496; RRID: AB_10013483
Rabbit Anti-tRFP-CF594	Biotium	Cat# 20422; RRID: AB_2686890
Mouse Anti-HA-594	Thermo Fisher	Cat# A-21288; RRID: AB_2535830
Mouse Anti-HA -647	Cell Signaling	Cat# 3444S; RRID: AB_10693329
Mouse Anti-V5 -488	AbDSerotec	Cat# MCA1360A488; RRID: AB_770155
Guinea Pig anti-mKate2	Dawen Cai - University Michigan	N/A
Rabbit anti tRFP/Kate2	Evrogen	Cat# AB233; RRID: AB_2571743
Biotinilated Isolectin B4	Vector	Cat# B-1205; RRID: AB_2314661
Rabbit anti-ERG	Abcam	Cat# ab110639; RRID: AB_10864794
Rabbit anti-Sox2	Millipore	Cat# AB5603; RRID: AB_2286686
Rat anti-mouse VE-cadherin	BD Biosciences	Cat# 555289; RRID: AB_395707
Rabbit Anti-PhiYFP	Evrogen	Cat# AB602
Rabbit anti-Phospho-p44/42 MAPK (ERK)	Cell Signaling	Cat# 4370; RRID: AB_2315112
Mouse Anti-B-Tubulin III (neuronal)	Sigma	Cat# T8578; RRID: AB_1841228

**Chemicals, Peptides, and Recombinant Proteins**		

Hoechst 33342	Invitrogen	H1399

**Critical Commercial Assays**		

Click-it EdU Alexa Fluor 647 Imaging kit	Thermo Fisher	C10340
High Capacity cDNA kit	Thermo Fisher	4368814
Mouse Gene Expression Taqman assays	Thermo Fisher	Diverse Codes
Taqman Universal PCR Master Mix	Thermo Fisher	4304437

**Experimental Models: Cell Lines**		

E. Coli XL1 Blue - Ultracompetent	Agilent	#200249
E. Coli EL250	CNIC	Lee et al., 2001
G4 Mouse ES cells	Andreas Nagy	George et al., 2007
G4 Mouse ES cells: *iMb-Control-Mosaic Gt(Rosa)26Sor*^*tm1(iMb-Control-Mosaic)*^	This Paper	N/A
G4 Mouse ES cells: *iMb-Notch-Mosaic*(*Gt(Rosa)26Sor*^*tm1(iMb-Notch-Mosaic)*^*)*	This Paper	N/A
G4 Mouse ES cells: *iMb-Vegfr2-Mosaic*	This Paper	N/A
G4 Mouse ES cells: *iChr-Control-Mosaic*	This Paper	N/A
G4 Mouse ES cells: *iChr-Notch-Mosaic*	This Paper	N/A
G4 Mouse ES cells: *iChr-Notch-v2-Mosaic*	This Paper	N/A
G4 Mouse ES cells: *iMb2-Control-Mosaic*	This Paper	N/A
G4 Mouse ES cells: *iChr2-Control-Mosaic*	This Paper	N/A
G4 Mouse ES cells: *iChr2-Notch-Mosaic*	This Paper	N/A
G4 Mouse ES cells: Dual iMb x iChr Mosaic	This Paper	N/A
G4 Mouse ES cells: Dual iMb2 x iChr2 Mosaic	This Paper	N/A
G4 Mouse ES cells: Acceptor ES cells for ifgMosaic targeting in pre-modified Rosa26 locus	This Paper	N/A
OP9 cells	ATCC	CRL-2749

**Experimental Models: Organisms/Strains**		

Mouse: *iMb-Control-Mosaic* (*Gt(Rosa)26Sor*^*tm1(iMb-Control-Mosaic)*^*)*	This Paper	N/A
Mouse: *iMb-Notch-Mosaic*(*Gt(Rosa)26Sor*^*tm1(iMb-Notch-Mosaic)*^*)*	This Paper	N/A
Mouse: *iMb-Vegfr2-Mosaic* (*TgBAC(Rosa)26Sor*^*(iMb-Vegfr2-Mosaic)*^*)*	This Paper	N/A
Mouse: *iChr-Control-Mosaic* (*Gt(Rosa)26Sor*^*tm1(iChr-Control-Mosaic)*^*)*	This Paper	N/A
Mouse: *iChr-Notch-Mosaic* (*Gt(Rosa)26Sor*^*tm1(iChr-Notch-Mosaic)*^*)*	This Paper	N/A
Mouse: *iChr-Notch-v2-Mosaic* (*TgBAC(Rosa)26Sor*^*(iChr-Notch-v2-Mosaic)*^*)*	This Paper	N/A
Mouse: *iMb2-Control-Mosaic* (*Tg(Rosa)26Sor*^*(iMb2-Control-Mosaic)*^*)*	This Paper	N/A
Mouse: *iChr2-Control-Mosaic* (*Gt(Rosa)26Sor*^*tm1(iChr2-Control-Mosaic)*^	This Paper	N/A
Mouse: *iChr2-Notch-Mosaic* (*Gt(Rosa)26Sor*^*tm1(iChr2-Notch-Mosaic)*^	This Paper	N/A
Mouse: *Tie2-Cre*	CNIC	Kisanuki et al., 2001
Mouse: *Cdh5(PAC)-CreERT2*	CNIC	Wang et al., 2010
Mouse: *Polr2a-CreERT2*	CNIO/CNIC	Guerra et al., 2003

**Oligonucleotides**		

See Table S1	Sigma	N/A

**Recombinant DNA**		

pmKate2-H2B	Evrogen	FP311
CMV-Brainbow-1.1 M	Livet et al., 2007	Addgene # 18722
pThy1-Brainbow3.2	Cai et al., 2013	Addgene # 45179
p3-H2B-Cherry	Nadia Mercader Lab	N/A
Mb2-HA-Tfp1	Genscript DNA synthesis	N/A
pCAG:H2B-EGFP	Hadjantonakis and Papaioannou, 2004	Addgene # 32599
pLVX-Tight-Puro	Clontech	N/A
pcDNA3.1	Invitrogen	N/A
piiTRE-Bi-SG-Tii	Li et al., 2010	Addgene # 26121
Vegfr2 active form	Kurt Ballmer-Hofer Lab	Dosch and Ballmer-Hofer 2010
Rosa26 BAC	*bacpac.chori.org*	RP23-401D9
pRosa26	Philipe Soriano	Soriano, 1999
iMb2-Control-Mosaic (SO273)	This paper, Figure S6	Addgene # 99750
iChr2-Control-Mosaic (SO274)	This paper, Figure S6	Addgene # 99751
iChr2-Notch-Mosaic (SO250)	This paper, Figure S6	Addgene # 99752
iMb-Control-Mosaic (IR98.10)	This paper, Figure S2	Addgene # 99748
iMb-Notch-Mosaic (IR99.40)	This paper, Figure S2	Addgene # 99749
LoxP1-Mb2YFP-2A (SO244)	This paper, Figure S7	Addgene #99613
LoxP2-Mb2Tomato-2A (SO240	This paper, Figure S7	Addgene #99614
LoxP3-Mb2-HA-mTFP1-2A (SO157)	This paper, Figure S7	Addgene #99615
LoxP1-His-H2B-Cherry-2A (SO290)	This paper, Figure S7	Addgene #99616
LoxP2-H2B-EGFP-V5-2A (SO107)	This paper, Figure S7	Addgene #99617
LoxP3-HA-H2B-Cerulean-2A (SO104)	This paper, Figure S7	Addgene #99618
FRT1-Mb2YFP-2A (IG153)	This paper, Figure S7	Addgene #99619
FRT2-Mb2Tomato-2A (IG154)	This paper, Figure S7	Addgene #99620
FRT3-Mb2-HA-mTFP1-2A (IG155)	This paper, Figure S7	Addgene #99621
FRT1-His-H2B-Cherry-2A (IG156)	This paper, Figure S7	Addgene #99622
FRT2-H2B-EGFP-V5-2A (IG157)	This paper, Figure S7	Addgene #99623
FRT3-HA-H2B-Cerulean-2A (IG158)	This paper, Figure S7	Addgene #99624
Triple ORF LoxP ifgMosaic Donor (LH208)	This paper, Figure S7	Addgene #99625
Triple ORF FRT ifgMosaic Donor (IG135)	This paper, Figure S7	Addgene #99626
pRosa26-4x-Insulator (JP6)	This paper, Figure S7	Addgene #99627
pX330 Rosa26 AscI LH291 Gu1 (LH500)	This paper, Figure S7	Addgene #99628
pX330 Rosa26 Gu1 (LH416)	This paper, Figure S7	Addgene #99629
Tre-Tight ifgMosaic donor Vector (LH121)	This paper, Figure S7	Addgene #99630
4xnr UAS ifgMosaic donor Vector (IG230)	This paper, Figure S7	Addgene #99631
pRosa26-Acceptor (AG103)	This paper, Figure S7	Addgene #99632
BAC Rosa26 iMb-Vegfr2-Mosaic (G273)	This paper, Figures 3, S2, and S5	N/A
BAC Rosa26 iChr-Notch-V2-Mosaic (G280)	This paper, Figures S2 and S5.	N/A
BAC Rosa26 iMb VEGFR3/VEGFR2 Mosaic (G234)	This paper, Figure S5	N/A
BAC Rosa26 iMb-Notch-V2-Mosaic (G203)	This paper, Figure S5	N/A
BAC Rosa26 iMb-Notch-V3-Mosaic (G210)	This paper, Figure S5	N/A
BAC Rosa26 iMb-Control-V2-Mosaic (G208)	This paper, Figure S5	N/A
BAC Rosa26 iChr-Control-V2-Mosaic (G284)	This paper, Figure S5	N/A
BAC Rosa26 4xnrUAS-iMb-Notch-Mosaic (G267)	This paper, Figure S5	N/A
BAC Rosa26 4xnrUAS-iMb-Vegfr2-Mosaic (G276)	This paper, Figure S5	N/A
BAC Rosa26 Tre-Tight-iMb-VEGFR3/2 Mosaic (G237)	This paper, Figure S5	N/A
BAC Rosa26 CAG Acceptor (G226)	This paper, Figure S7	N/A
BAC Rosa26 Tre-Tight Acceptor (G228)	This paper, Figure S7	N/A
BAC Rosa26 4xnrUAS Acceptor (G256)	This paper, Figure S7	N/A

**Software and Algorithms**		

DNAstar SeqBuilder software	Lasergene	https://www.dnastar.com/t-seqbuilder.aspx
Fiji	Schindelin et al., 2012	https://imagej.net/Fiji
Leica SP5 and SP8 Confocal Software	Leica	http://www.leica-microsystems.com
ZEISS LSM700 and LSM780 Confocal software	Zeiss	https://www.zeiss.com/global/home.html

### Contact for Reagent and Resource Sharing

Further information and requests for reagents should be directed to and will be fulfilled by Lead Contact, Rui Benedito (Rui.benedito@cnic.es).

### Experimental Model and Subject Details

#### Animals

To generate *Rosa26* gene-targeted mice (*Mus musculus*) a G4 mES cell line with high germline transmission potential (George et al., 2007) was used to perform *Rosa26* gene targeting with different *ifgMosaic* constructs by the institute gene targeting facility using standard methods. To generate transgenic mice containing the *Rosa26 ifgMosaic* BAC, 2ng/ul of the BAC Maxiprep was co-injected with 100ng/ul of HyPBase transposase RNA in the pronucleus of fertlized mouse eggs by the host institute transgenesis service. Several mouse lines (Figures S2, S5 and S6) were generated for this study, and images of nine of these lines are shown in this study. This includes mouse lines *iMb-Control-Mosaic* (*Gt(Rosa)26Sor*^*tm1(iMb-Control-Mosaic)*^*)*; *iMb-Notch-Mosaic* (*Gt(Rosa)26Sor*^*tm1(iMb-Notch-Mosaic)*^*)*; *iMb-Vegfr2-Mosaic* (*TgBAC(Rosa)26Sor*^*(iMb-Vegfr2-Mosaic)*^*)*; *iChr-Control-Mosaic* (*Gt(Rosa)26Sor*^*tm1(iChr-Control-Mosaic)*^*)*; *iChr-Notch-Mosaic* (*Gt(Rosa)26Sor*^*tm1(iChr-Notch-Mosaic)*^*); iChr-Notch-v2-Mosaic* (*TgBAC(Rosa)26Sor*^*(iChr-Notch-v2-Mosaic)*^*); iMb2-Control-Mosaic* (*Tg(Rosa)26Sor*^*(iMb2-Control-Mosaic)*^*); iChr2-Control-Mosaic* (*Gt(Rosa)26Sor*^*tm1(iChr2-Control-Mosaic)*^) and *iChr2-Notch-Mosaic* (*Gt(Rosa)26Sor*^*tm1(iChr2-Notch-Mosaic)*^). To induce the genetic mosaic in ECs we used the transgenic mouse lines *Tie2-Cre* (Kisanuki et al., 2001) or *Cdh5(PAC)-CreERT2* (Wang et al., 2010). To induce the genetic mosaics in the neural tube we used the *Polr2a-CreERT2* mouse line (Guerra et al., 2003). To activate recombination in animals containing these *CreERT2* alleles, tamoxifen (Sigma) was injected in pups at the indicated stages (35μg/g) or in combination with progesterone in pregnant adult females (2mg tamoxifen and 1mg progesterone). Male and female mice were used for the analysis, which were maintained under specific pathogen-free conditions. Genotyping primers are provided in Table S1. Experiments involving animals were conducted in accordance with institutional guidelines and laws, following protocols approved by local animal ethics committees and authorities.

### Method Details

#### ifgMosaic Recombinant DNA

The basic elements of the different *ifgMosaic* constructs were obtained from different sources and assembled by standard DNA cloning methods. The unique restriction sites and the sequential modular cloning strategy were designed and selected by using the DNAstar SeqBuilder software (Lasergene) and previous experience with commercially available restriction enzymes. The TdTomato DNA sequence was obtained by PCR from genomic DNA of the *Rosa26 mT/mG* mouse line (Muzumdar et al., 2007). mKate2 sequence was obtained from the pmKate2-H2B vector (FP311, Evrogen). Cerulean, EYFP, N-PhiMut, modified *loxP* sites and the sv40 polyadenylation sequences were obtained from Addgene plasmids CMV-Brainbow-1.1 M and pThy1-Brainbow3.2, a gift from Joshua Sanes (Addgene plasmids # 18722 and # 45179). H2B-Cherry was obtained from p3-H2B-Cherry (a gift from Nadia Mercader). Mb2-HA-Tfp1 or *FRT* sites containing cassettes were synthesized at Genscript. The 41 aminoacids coding the Mb2 tag sequence of this construct was subsequently subcloned by PCR and fused to EYFP, EGFP and TdTomato. The H2B tag and EGFP were obtained from pCAG:H2B-EGFP a gift from Anna-Katerina Hadjantonakis & Virginia Papaioannou (Addgene plasmid # 32599). The WPRE and Tre-Tight sequences were obtained from pLVX-Tight-Puro (Clontech). The bGH polyadenylation sequences were obtained from pcDNA3.1 (Invitrogen). The 2A peptide sequence of the *Thosea asigna virus* (TaV) was subcloned by PCR. The insulator sequences were obtained from plasmid piiTRE-Bi-SG-Tii, a gift from Liqun Luo (Addgene plasmid # 26121). The *Cdh5* gene short 3' homology sequence was obtained by PCR. The VEGFR2 active form, with a deletion of the extracelular domain, was cloned from *pcDNA3_hVegfr2-DeltaEC-V6E* vector, a gift from Kurt Ballmer-Hofer. The Vegfr2^TK−^ version was obtained by cDNA PCR amplification and mutagenesis (Y1173F) of the mouse gene *Vegfr2 (Kdr)*. The *DN-Maml1* and *NICD-PEST* sequences were subcloned by PCR from mouse cDNA and following previous work (Maillard et al., 2004). The gene targeting construct used to generate the i*fgMosaic* mouse lines obtained by classical *Rosa26* gene targeting, is a modified version of the plasmid pRosa26 (Soriano, 1999). It includes a smaller backbone and several rare restriction sites flanking the homology arms, that due to their longer sequence, are unlikely to cut any other subcloned cassete or gene of interest. These are used to linearize and remove the vector backbone of the large *ifgMosaic* plasmids before classical or Cas9 assisted *Rosa26* gene targeting.

To generate the BAC transgenic *ifgMosaic* constructs and mice, we modified an existing BAC (RP23-401D9) containing 190kb of the mouse *Rosa26* locus. In the backbone of this BAC we introduced the PiggyBAC transposon elements flanked by an Ampicillin resistance gene expressing cassette (Rostovskaya et al., 2013), to select for recombinant BACs in EL250 bacteria. Later we introduced by DNA recombineering the assembled cassette *INS-INS-CAG-Promoter-L1L2L3-N-PhiM-pA-3-homology-FRT-PGK-Neo-FRT-INS-INS*, that allow us later to introduce any donor *ifgMosaic* construct containing up to 3 different ORFs flanked by three different *loxP* sites. For the method used to clone the genes of interest and assemble all the different modular constructs into the *ifgMosaic* donor vector see Figures 5, 7, S4 and S7. Plasmids generated for this study were deposited at Addgene (accession numbers in key resources table). Bacterial clones containing the BACs used in this study are available upon request.

#### BAC recombineering and preparation for microinjection

The preparation of electrocompetent EL250 cells, transformation with the *Rosa26* BAC and recombineering was carried out according to previous protocols (Lee et al., 2001). After obtaining EL250 bacteria containing the acceptor *Rosa26* BAC we induced homologous recombination with donor vectors by heat activating the EL250 lambda prophage recombination machinery after electroporation. Briefly, the bacterial culture was grown overnight at 30°C in Luria–Bertani (LB) broth without salt and with chloramphenicol (12.5 μg/ml). In a 250 ml conical flask containing 25ml of LB broth we diluted (1:50) the overnight culture and incubated at 30°C until reaching an OD_600_ of 0.2. Then, 12 ml of culture were transferred to a pre-warmed 250 ml conical flask and heat-shocked at 42°C for 10 min in a shaking incubator (induced sample). The remaining culture was kept as the non-induced control. The two samples, induced and non-induced, were cooled down in ice for 10 min and then transferred to two 15 ml Falcon tubes and pelleted by centrifugation (1000g at 4°C for 8 min). The supernatants were poured off and the pellets were gently resuspended in 1 ml ice-cold Milli Q water. Subsequently, the samples were transferred to 1.5 ml tubes and were spun down using 20000g for 20 sec. Then, pellets were washed with 1ml of Milli Q water twice. Finally, the pellets were resuspended in 40 μl of 10% glycerol in water and stored on ice. 200 ng of linearized *ifgMosaic* Donor vector (maximum volume of 10 ul) were added to the non-induced and induced samples, and then up to 50ul of the mixes were transferred in a 0.1 cm cuvette for electroporation (BioRad, California, USA). A voltage of 1.8 (V/cm^3^), and an electric pulse of 1.5 to 5 (msec) was used. Immediately after, the transformed bacterial cells were incubated in 500 μl of LB for 1 h at 30°C. 100 μl of each transformation was spread out in LB plates supplemented with chloramphenicol (12,5μg/ml) and kanamycin (12,5μg/ml) for selection of the bacteria containing the recombinant *Rosa26 ifgMosaic* BACs. The number of colonies obtained in the induced versus the non-induced plates was on average between 10 to 50 times higher. Single colonies were screened, by BAC DNA Miniprep and restriction analysis with PmeI/AscI enzymes. The acceptor *Rosa26* BAC DNA was used as negative control. The *FRT-PGK-Neo-FRT 3’ homology-FRT* cassette was removed by inducing the expression of the *FLPe* gene present in EL250 bacteria with arabinose. For this purpose, positive colonies were grown up to O.D._600_=0.8 in LB. Then, L-arabinose was added to the culture to get a final concentration of 0.2%, and bacteria were incubated at 30°C for 1 h. After that the culture was diluted 1:10 and incubated at 30°C for 2 h. 1ul of the culture was plated in a chloramphenicol (12.5 μg/ml) LB plate and 10ul of the culture were plated in a kanamycin (12.5 μg/ml) LB plate (as negative control). The number of colonies in the Chloramphenicol plate was much higher than in the Kanamycin plates, confirming that most bacteria had recombined the Neo/Kanamycin resistance cassette. To further confirm the structure of the flipped *Rosa26* BAC after the entire procedure, we performed a final AscI/PmeI restriction digestion analysis that allows us to confirm the integrity of the *ifgMosaic* construct inserted in the *Rosa26* BAC. A single colony containing the final flipped *Rosa26 ifgMosaic* BAC was grown overnight in PXYT media at 30°C. A modified Qiagen Maxiprep protocol (Appendix A within the Qiagen Maxiprep protocol) was used to obtain on average around 200-500ng/ul of BAC DNA in 100ul of water or microinjection buffer. This solution was kept at 4°C for not more than one month before microinjection.

#### ES cell culture and CRISPR/Cas9 gene targeting in pre-modified Rosa26 locus

Mouse ES cells with the G4 background (George et al., 2007) were cultured in standard ES cell media (DMEM containing Glutamax (31966-047, Gibco), 15% FBS (tested for germline transmission), 1 x NEAA (Hyclone, SH3023801), 0.1% ß-mercaptoethanol (Sigma, M7522), 1 x Pen/Strep (Lonza, DE17-602E) and LIF) in dishes covered with a feeder layer of mouse embryonic fibroblasts (MEFs). For classical gene targeting of large plasmids, 25ug of linearized DNA was used to electroporate 5 million cells. Selection in 200ug/ml G418 (Geneticin) was performed for 6 days, after which individual colonies were picked for storage, PCR and Southern blot screening. Selected positive clones were expanded and used for microinjection in host blastocysts of the C57Bl/6J strain. Chimeras with high percentage of agouti coat color were then crossed with mice to obtain germline transmission of the targeted insertion. To generate the *ifgMosaic* acceptor mouse ES cell line, we first used the same standard gene targeting procedure mentioned above and the *ifgMosaic* recipient vector that contains a *FRT-PGK-Neo-FRT* cassette for selection (Figure 6B). Afterwards we used transient transfection with a Flpo expressing plasmid to remove the *FRT-PGK-Neo-FRT* cassette. This allowed us to perform additional targetings of the pre-modified *Rosa26* locus that contains all the necessary regulatory elements and an unique sequence for DNA double-strand break (DSB) with Cas9 (Figure 6B). To generate *Rosa26 ifgMosaic* ES cell lines we used this modified G4 ES cell line and performed nucleofection (Amaxa) with two plasmids. One expresses the sgRNA and the Cas9 protein (*px330_U6_Gu1 Rosa26_CBh_hSpCas9*) and the other is a donor *ifgMosaic* plasmid that will be used as template for DSB repair by homologous recombination. For each nucleofection we ressuspended 2.5 million ES cells in 100ul volume containing 1.5ug of circular px330 plasmid and 3.5ug of the circular donor plasmid. After nucleofection we plated 5ul or 30ul of the mix in two different wells on a 6-well plate with MEFs. Six days after G418 selection, 12 isolated ES cell colonies were picked for storage and further screening. PCR with the flanking primers GTGGGCTCTATGGCTTCTGA and ACTCCAGGACGGAGTCAGTG, allowed us to identify ES cell clones with precise homologous recombination and insertion of the *ifgMosaic* cassette. After identification of clones with precise gene targeting, we further validated the *ifgMosaic* functionality by transfecting the ES cells with a Cre expressing plasmid. Strong expression of the 3 FPs in different cells further confirmed the *ifgMosaic* gene targeting and screening method since the donor plasmid does not contain the CAG enhancer, and it will be only expressed after proper gene targeting in the pre-modified *Rosa26* locus.

#### Embryoid Bodies and endothelial differentiation

To generate embryoid bodies (EBs) from ES cells we used the standard hanging drop method. Briefly ES cells were first grown for 2 days on gelatinized plates and after, tripsinized and ressuspended at a density of 60.000 cells per ml in embryoid bodies media (DMEM Glutamax (Gibco, 31966-047), 15% FBS, HEPES (Biowhittaker EE17-737) and Monotyoglycerol (Sigma, M6145). For each EB, 20ul drops of this solution were pipetted onto the lid of a petri dish. This lid was inverted, to form the hanging drops, and the dish further filled with PBS to prevent evaporation. Four days after differentiation the embryoid bodies were plated on an OP9 cells monolayer and differentiated in basal media (MEM alpha (Gibco, 11900-016), supplemented with 20% FBS and 7.5% Sodium bicarbonate (Gibco, 25080-060)) containing 30ng/ml of VEGF to further induce endothelial differentiation and proliferation for 5 days.

#### Immunostainings

For immunostaining of ES cells or ECs derived from embryoid bodies, cells were fixed for 10 minutes in PBS containing PFA4% and Sucrose 4%. After a brief rinse in PBS, cells were permeabilized in 0.1% Triton for 10 minutes and then immersed in a blocking solution (10% Fetal bovine serum in PBS). Primary antibodies (see Key Resources Table in STAR Methods) were diluted in blocking solution and incubated for 2 hours at room temperature or overnight, followed by three washes in PBS of 10 minutes each and incubation for 1 to 2 hours with conjugated secondary antibodies (Invitrogen or Biotium) at room temperature. After three washes in PBS, cells were mounted with Fluoromount-G (SouthernBiotech).

For immunostaining of mouse retinas, eyes from mouse pups were dissected and fixed for 1 hour in a solution of PFA4% in PBS. After washing the tissue in PBS twice, retinas were microdissected and processed for immunostaining following a very similar protocol previously described above for the cells. The only difference is that the blocking/permeabilization buffer contains 0.3% Triton, 3% FBS, 3% Donkey Serum and antibody washes were more extended in time; on average for 30 minutes each.

#### FACS and qRT-PCR analysis

For routine analysis of fluorescent protein ratios obtained in ES cells we lipofected them with Cre expressing plasmids and 3 days after collected the cells for FACSAria analysis. In some cases these ES cells were differentiated to embryoid bodies and endothelial cells, as shown above, and after trypsinization, cells were analysed in a FACSAria containing lasers to detect Cerulean/Tfp1, GFP/YFP and Tomato/Cherry.

For the qRT-PCR analysis, endothelial cells were isolated from E10.5 mouse embryos or postnatal day 20 mouse hearts. Cells were immunolabelled with CD31-APC antibody and separated, according to their fluorescence, in a FACSAria. Cells were sorted directly to RNeasy Mini Kit RLT buffer (Qiagen). RNA was extracted according to the Qiagen protocol. cDNA was synthetized with the High Capacity cDNA kit from Applied Biosystems (AB). cDNA was quantified by qRT-PCR with Taqman assays and universal master mix on a AB 7900 qRT-PCR machine.

#### Microscopy

Depending on the complexity of the immunostainings and the combination of FPs to detect, we used different laser-scanning confocal microscopes. For up to 4 channels acquisition of large fields we used the ZEISS LSM700 inverted microscope with laser lines 405, 488, 546 and 633nm. For multi-color detection of up to 7 different signals we used the inverted Leica SP5 confocal (405, 488, 514, 546, 594, 633nm) or the Leica SP8 confocal with a 405nm laser and a white laser that allows excitation at any wavelength from 470nm to 670nm. Occasionally, a ZEISS LSM780 with a GaAsP spectral detector was used. To record the Movies S1 and S2, we used a Leica SP5 confocal with a sensitive hybrid detector and with an incubation chamber for temperature (37°C) and CO2 control (5% CO2). We used multi-well slides (IBIDI) containing the ES cells growing on top of a monolayer of MEFs and a 20x multi-immersion objective covered with glycerol. Tile scan and volume (3D) acquisition was performed every 15 minutes for a period of 16 hours. For the mouse retina laser scanning confocal analysis we used the 10x, 20x or 40x lens. Individual fields or tiles of large areas were acquired.

### Quantification and Statistical Analysis

#### Selection and quantification of mosaic clone size and dispersion

In ES cells transfected with Cre, the different cellular ratios were obtained by FACS or after immunostaining for the different fluorescent proteins (anti-GFP, anti-Dsred, anti-Kate2) or specific epitopes (V5 or HA tag). Quantification was performed with Fiji/ImageJ. Signal tresholds were defined before quantification of the number of cells/nuclei having a specific color. In the case of mosaics of cells expressing only membrane localized FPs, relative areas were quantified and related with cell number, after quantifying the average cell number per area based on Hoechst staining or nuclei marker proteins.

In the neural tube of E12.5 embryos, clones were identified on 3D confocal scanning volumes (4-5 Z slices), acquired from 20μm thick cryosections, after immunostaining for the different marker proteins (Fig. 2C and S3B). Clones were scored based on their nuclei colour, number and distribution. Sections with too many clones having the same colour were not quantified. In the neural tube, neuronal progenitors obey horizontal domains (Briscoe and Small, 2015), being easier to assign single-cell derived progeny.

In the retinas of newborn mice, a superficial network of vessels grows from P1 to P7. Retinas can be microdissected, stained and flat-mounted with this superficial network facing the coverslip. Endothelial clones were identified on large 3D tile confocal scanning volumes (2-4 Z slices), acquired from wholemount fixed retinas, stained for different marker proteins. 20x or 40x objectives were used for the tile scanning and Fiji/ImageJ to threshold, select and quantify clones. For the selection of clones in these volumes different parameters were considered. The clone single or dual color-code, its relative intensity (which varies between clones derived from different cells), its size and dispersion, and its proximity to other clones. To calculate the average Dual *ifgMosaic* clone dispersion, relative to its size, we measured in Fiji/ImageJ, the shortest path linking the center of the identified clone nuclei (see Figure S3F). For the accurate quantification and delimitation of the most frequent dual clones, in areas with higher frequencies of recombination, the average clone dispersion value can be used to define areas that are very likely to contain all cells of an individual clone and no cells from adjacent single-cell derived dual clones. The higher the signal intensity and spectral separation, the easier is the quantification process. With the second generation *ifgMosaic* mice automatic signal data segmentation and quantification can be applied.

#### P-ERK relative signal intensity quantification

The average background level of P-ERK immunofluorescence signal was quantified in the non-vascular tissue surrounding IsolectinB4+ ECs and assigned as zero intensity value (Fig. 3D). Individual IsolectinB4+/MbTomato+ and adjacent IsolectinB4+/MbTomato- ECs were selected based on the anti-dsred immunofluorescent signal. Within the MbTomato- endothelial population, tip cells were manually selected according to their position at the edge of the angiogenic front, and stalk-cells were selected as non-tip cells adjacent to MbTomato+ cells. The average absolute pixel P-ERK signal intensity was quantified in each selected cell area, in relation to the average non-vascular signal background level.

#### Statistical analysis

In the case of two groups comparisons, two-tailed, Student's T test was used. ANOVA was used for multiple groups comparison. In the case of the nonparametric data displayed in Figure 4G, Kruskal-Wallis one-way analysis of variance was used. p values above 0,05 were considered not significant (NS). All calculations and charts were performed with GraphPad Prism software. No randomization or blinding was used and animals/tissues were selected for analysis based on the detected Cre-dependent recombination frequency and quality of multiplex immunostaining. The sample size was chosen according to the observed statistical variation and published protocols.

### Data and Software Availability

The DNA sequences of the plasmids used in this study, and illustrated in Figures S2, S6, and S7, are deposited at Addgene (#99613–99632 and #99748–99752).

## Author Contributions

L.H., A.G.-S., I.R.-A, J.R.P., S.F.R., and S.D.O.-C. generated plasmids and BAC constructs. S.P.-Q., L.H., A.H., M.F.-C., W.L., and R.B. analyzed the recombination and expression of the constructs in the different mouse lines. G.G., V.C.-G., I.G.-G., M.B., and R.B. generated and analyzed mouse ES cell lines with classical or CRISPR-Cas9 gene-targeting techniques. L.M.C.-R. gave expert advice and generated the mouse lines by BAC transgenesis in fertilized eggs or blastocyst injection of modified ES cells. M.S.S.-M. genotyped and organized the mouse colonies. S.P.-Q., L.H., M.F.-C., W.L., I.G.-G., and R.B. designed experiments and interpreted results. R.B. and S.P.-Q. wrote the manuscript.

## Supplemental Information

Table S1. Genotyping Oligonucleotides

Movie S1. HEK cells expressing different combinations of dual reporter constructs, Related with Figure S1

Movie S2. Mouse ES cells expressing the *Dual ifg2Mosaic*, Related with Figure 7
