# Supplementary material for: Dual ifgMosaic: A Versatile Method for Multispectral and Combinatorial Mosaic Gene-Function Analysis
Source: Cell. 2017 Aug 10;170(4):800–814.e18. doi: 10.1016/j.cell.2017.07.031 (PMC6381294; doi:10.1016/j.cell.2017.07.031)
Supplement: Table S1. Genotyping Oligonucleotides [file mmc1.docx]

**Table S1:**

| Genotyping Oligonucleotides | Source |
| --- | --- |
| Tie2 T5: GGGAAGTCGCAAAGTTGTGAGTT | Sigma |
| Tie2 C2: CTAGAGCCTGTTTTGCACGTTC | Sigma |
| Rosa26 RR711: GCACTTGCTCTCCCAAAGTC | Sigma |
| Rosa26 RR712: GGGCGTACTTGGCATATGAT | Sigma |
| Rosa26 RR713: CTTTAAGCCTGCCCAGAAGA | Sigma |
| Rosa26 RR714: GCGAAGAGTTTGTCCTCAACC | Sigma |
| Rosa26 NEW 1459: CGGGGTCATTAGTTCATAGCC | Sigma |
| Rosa26 NEW 1460: CACCTCGACCATGGTAATAGC | Sigma |
| iChr Phi F: ACGTGAAGCTGAGCAAGGAT | Sigma |
| iChr H2B R: CTTAGTCACCGCCTTCTTGG | Sigma |
| hVEGFR2 seq F: GCGGCACGAAATATCCTCT | Sigma |
| hVEGFR2 seq R: ATTTCCCACAGCAAAACACC | Sigma |
| Cdh5 Trans F: GGAGGCTGGAAAGTAGAGCA | Sigma |
| CreM R: TCCCTGAACATGTCCATCAG | Sigma |
